# AKR1C1 controls cisplatin-resistance in head and neck squamous cell carcinoma through cross-talk with the STAT1/3 signaling pathway

**DOI:** 10.1186/s13046-019-1256-2

**Published:** 2019-06-10

**Authors:** Wei-Min Chang, Yu-Chan Chang, Yi-Chieh Yang, Sze-Kwan Lin, Peter Mu-Hsin Chang, Michael Hsiao

**Affiliations:** 1 Genomics Research Center, Academia Sinica, Taipei, Taiwan; 2 Department of Dentistry, National Taiwan University Hospital, Taipei, Taiwan; 3 Department of Oncology, Taipei Veterans General Hospital, Taipei, Taiwan; 4 Faculty of Medicine, College of Medicine, National Yang-Ming University, Taipei, Taiwan; 5 Department of Biochemistry, Kaohsiung Medical University, Kaohsiung, Taiwan

**Keywords:** AKR1C1, Cisplatin-resistance, HNSCC, STATs, Ruxolitinib

## Abstract

**Background:**

Cisplatin is the first-line chemotherapy used against most upper aerodigestive tract carcinomas. In head and neck cancer, sensitivity to cisplatin remains the key issue in treatment response and outcome. Genetic heterogeneity and aberrant gene expression may be the intrinsic factors that cause primary cisplatin-resistance.

**Methods:**

Combination of the HNSCC gene expression data and the cisplatin sensitivity results from public database. We found that aldo-keto reductase family 1 member C1 (AKR1C1) may be associated with cisplatin sensitivity in HNSCC treatment of naïve cells. We examined the AKR1C1 expression and its correlation with cisplatin IC_50_ and prognosis in patients. The in vitro and in vivo AKR1C1 functions in cisplatin-resistance through overexpression or knockdown assays, respectively. cDNA microarrays were used to identify the upstream regulators that modulate AKR1C1-induced signaling in HNSCC. Finally, we used the cigarette metabolites to promote AKR1C1 expression and ruxolitinib to overcome AKR1C1-induced cisplatin-resistance.

**Results:**

AKR1C1 positively correlates to cisplatin-resistance in HNSCC cells. AKR1C1 is a poor prognostic factor for recurrence and death of HNSCC patients. Silencing of AKR1C1 not only reduced in vitro IC_50_ but also increased in vivo cisplatin responses and *vise versa* in overexpression cells. Cigarette metabolites also promote AKR1C1 expression. Transcriptome analyses revealed that STAT1 and STAT3 activation enable AKR1C1-induced cisplatin-resistance and can be overcome by ruxolitinib treatment.

**Conclusions:**

AKR1C1 is a crucial regulator for cisplatin-resistance in HNSCC and also poor prognostic marker for patients. Targeting the AKR1C1-STAT axis may provide a new therapeutic strategy to treat patients who are refractory to cisplatin treatment.

**Electronic supplementary material:**

The online version of this article (10.1186/s13046-019-1256-2) contains supplementary material, which is available to authorized users.

## Introduction

Cisplatin is the standard chemotherapeutic drug in head and neck squamous cell carcinoma (HNSCC) treatment [1] . Cisplatin causes platinum-DNA adducts that induce G2/S arrest and subsequent cell death in rapidly growing cancer cells. Furthermore, cisplatin also increases highly reactive mono- and biaquated cisplatin forms [2] and intracellular reactive oxygen species (ROS) levels following reaction with cytoplasmic proteins and biomolecules [3]. For treatment of naïve, locally advanced HNSCC patients, the initial cisplatin-based chemotherapy response can be up to 50% [4]. Half of the patients remain without response to cisplatin; furthermore, most patients will develop acquired cisplatin-resistance, which induces cancer recurrence. Cisplatin-resistance and recurrence are the major factors leading to cisplatin-based therapeutic failure in HNSCC patients [5]. Thus, it is important to understand the mechanism of cisplatin-resistance, which may enable the development of strategies that help patients to overcome chemoresistance and improve clinical outcome in HNSCC.

A tumor is a heterogeneous cell mixture which harbors various genetic mutations and diverse gene expression. Therefore, precision medicine has become a rising field in cancer therapy [6]. Recently, patient-derived tumor xenograft models function as accurate preclinical models to predict therapeutic response; however, they are labor intensive and expensive projects for the prediction of therapeutic outcome [7]. In contrast, a well characterized cancer cell line database such as Genomics of Drug Sensitivity in Cancer (GDSC) [8–10] could provide reliable information on chemotherapy drug-response, and gene expression profiles from the Cancer Cell Line Encyclopedia (CCLE) [11] could provide guidance in the search for novel resistance genes. Moreover, the expression level of candidate genes and their prognostic value can be examined in public microarray databases or TCGA cohorts of clinical cancer patients [12–14]. Using in silico analysis could assist researchers in elucidating the candidate genes accounting and provide a potential therapy niches for overcoming chemoresistance in HNSCC.

The mammalian hydroxysteroid dehydrogenases comprise four enzymes (AKR1C1-C4) that catalyze reduction of steroids and prostaglandins [15] and cluster on the chromosome 10p14–15 region. AKR1C1 and C2 are located on different strands of DNA but have highly similar (> 98%) protein coding sequences [16]. AKR1C1 and -C2 contribute 40% of the detoxification function of 4-methylnitrosamino-1-(3-pyridyl)-1-butanone (NNK) in tobacco-derived nitrosamine carcinogens [17] In this study, we analyzed the HNSCC cell gene expression profiles and inhibitory concentration (IC_50_) of cisplatin from the CCLE and GDSC databases. Interestingly, the AKR1C1 expression level was highly correlated to cisplatin IC_50_, and modulated AKR1C1 expression could affect the cisplatin response. Furthermore, the cigarette metabolites stimulate AKR1C1 expression in HNSCC. Using a JAK inhibitor will overcome AKR1C1 induced primary cisplatin-resistance. Here, we provided a novel, independent enzymatic mechanism of AKR1C1 through STAT3 activation for primary cisplatin-resistance in HNSCC.

## Materials and methods

### In silico analysis of cisplatin response and patient prognosis

The HNSCC gene expression profiles (GSE36133) in the CCLE database [11] were downloaded from Gene Expression Omnibus (GEO) and analyzed by Genespring GX software (Agilent). The HNSCC cisplatin IC_50_ data were downloaded from the GDSC database (release version 4, [10]). The TCGA HNSCC prognostic value and clinical characteristics of the recurrent HNSCC cohort were analyzed in SurvExpress or the CancerBrowser database and reformatted in GraphPad Prism or SPSS Software.

### Cell culture and reagents

Cell cultures were prepared and maintained according to a standard protocol. 293 T, FaDu, Cal-27, HSC-2, and HSC-4 cells were purchased from ATCC or JCRB cell bank and maintained according to the manufacturer’s instructions. Chemical reagents, vectors, and antibodies are listed in Additional file 1: Table S1. The cisplatin and 5-PBSA were prepared in sterile PBS or water and the ruxolitinib and cigarette metabolites, such as NAB, NAT, NNK and NNN, were prepared in DMSO.

### Cell viability assay and caspase activity assay

In the cisplatin viability assay, HNSCC cells (2 × 10^3^) were seeded in 96-well plates. After incubation overnight, the medium was replaced with 200 μl fresh medium containing various dosages of cisplatin, 5-PBSA or ruxolitinib for 72 h. At the endpoint, the medium was replaced with 200 μl fresh medium containing 30 μl AlamarBlue solution, then incubated an additional 4 h and measured for fluorescent intensity (Ex/Em: 560 nm/590 nm). In the caspase activity assay, stable cells were infected by pCT-Apoptosis-Luc virus and seeded in 6-well plates (2 × 10^5^ / well). Then, cells were incubated in the same conditions as previously described, but the caspase activity was measured by the One-Glo™ luciferase assay after cisplatin treatment at the IC_50_ for 24 h.

### Vector construction, gene expression and microarray assay

All primer sequences are listed in Additional file 1: Table S1. The AKR1C1 and AKR1C2 cDNA was purchased from DNASU and wild type and constitutive activation STAT1 and STAT3 were purchased from Addgene then recombined into pLenti6.3-DEST through gateway LR II recombinase. The enzymatic domain dead E127D clone was generated from AKR1C1 cDNA by site-direct mutagenesis. The AKR1C1 knockdown clones were purchased from RNAiCore (Taiwan). The AKR1C1 gene manipulation was performed as previously described [18]. AKR1C1 promoter region (− 1276 to + 0) was amplified from Cal-27 genomic DNA then cloned into HE cloning kit (Bio-tools, Taiwan) then confirmed sequence by Sanger sequencing. Then AKR1C1 promoter was subclone into SBI pGreenfire reporter vector. The AKR1C1 downstream genes and regulators in HNSCC were discovered by Affymetrix U133 microarray assays. The microarray analysis approach was analyzed as previously described [19]. Genes that were up- or downregulated with greater than 1.5-fold changes in response to AKR1C1 overexpression/knockdown were further subjected to computational simulation by Ingenuity Pathway Analysis (IPA; QIAGEN, Valencia, CA, USA) online tools to predict potential upstream regulators and the significant cellular pathways and functions. The microarray data were uploaded to the National Center for Biotechnology Information Gene Expression Omnibus (GEO, NCBI, GSE119444). The specific genes were validated by real-time CPR with EvaGreen-based qPCR assays.

### Western blot and real-time quantitative PCR (qPCR)

Western blot analyses and qPCR conditions were performed as previously described [20]. The antibody dilution conditions and primer sequences are listed in Additional file 1: Table S1.

### Cancer stem cell sphere formation assay

Stable cells (1 × 10^3^) were seeded in Corning Ultralow attachment 6-well plates with 2 mL sphere medium (50 mL DMEM with 20 ng/mL EGF, bFGF and 1 mL B27 supplement) and then incubated for 14 days to form cancer spheroids. Spheroids were stained with Hoechst 33342, and the spheroid numbers were measured by an ImageXpress Micro XLS HCS system. The spheroid number was counted only when cell number was above 50 cells.

### Animal studies

All animal experiments were performed in strict accordance with the recommendations in the guidelines for the Care and Use of Laboratory Animals of Academia Sinica. The protocol was approved by the Institutional Animal Care and Use Committee of the Genomic Research Center, Academia Sinica (Protocol No: AS-IACUC-18-03-1195). Male Nod-SCID gamma (NSG) mice aged 5–6 weeks were bred in the Genomic Research Center. The animals were housed in a climate-controlled room (12:12 dark-light cycle, with constant humidity and temperature) with food and water provided ad libitum. All efforts were made to minimize suffering. For the in vivo tumor burden assay, 5 × 10^6^ stable cells were resuspended in sterile phosphate-buffered saline (PBS), then injected subcutaneously (SC) into the right flank of the mice. Each group consisted of 5 animals. The tumor burden was measured with the following formula: tumor volume (V) = L × W × H. The mice were sacrificed, and the tumors were weighed and photographed. In in vivo cisplatin response assays, 2 mg/kg cisplatin were dissolved in PBS then injected through intraperitoneal injection.

### Statistical analysis

The association between cisplatin response and HNSCC gene expression level was analyzed by Pearson correlation coefficient. The HNSCC IC_50_ values were determined by the curve-fitting model with four-parameter logistic equation model in GraphPad Prism Software. An unpaired t-test was performed to compare the mRNA expression levels in different treatment groups. Estimates of the survival rates were calculated using the Kaplan-Meier method and compared using the log-rank test. Patient follow-up time was censored if the patient was lost during follow-up. For all experiments, bar graphs represent the mean (±SEM) from three independent experiments, and statistical analyses were performed using SPSS (Statistical Package for the Social Sciences) 21.0 software. Unless otherwise stated, significant differences between means were determined using a Student’s *t*-test. A *p* value of < 0.05 was considered significant for all of our analyses.

## Results

### AKR1C1 expression is correlated with cisplatin-resistance and clinical outcome

**Fig. 1:**
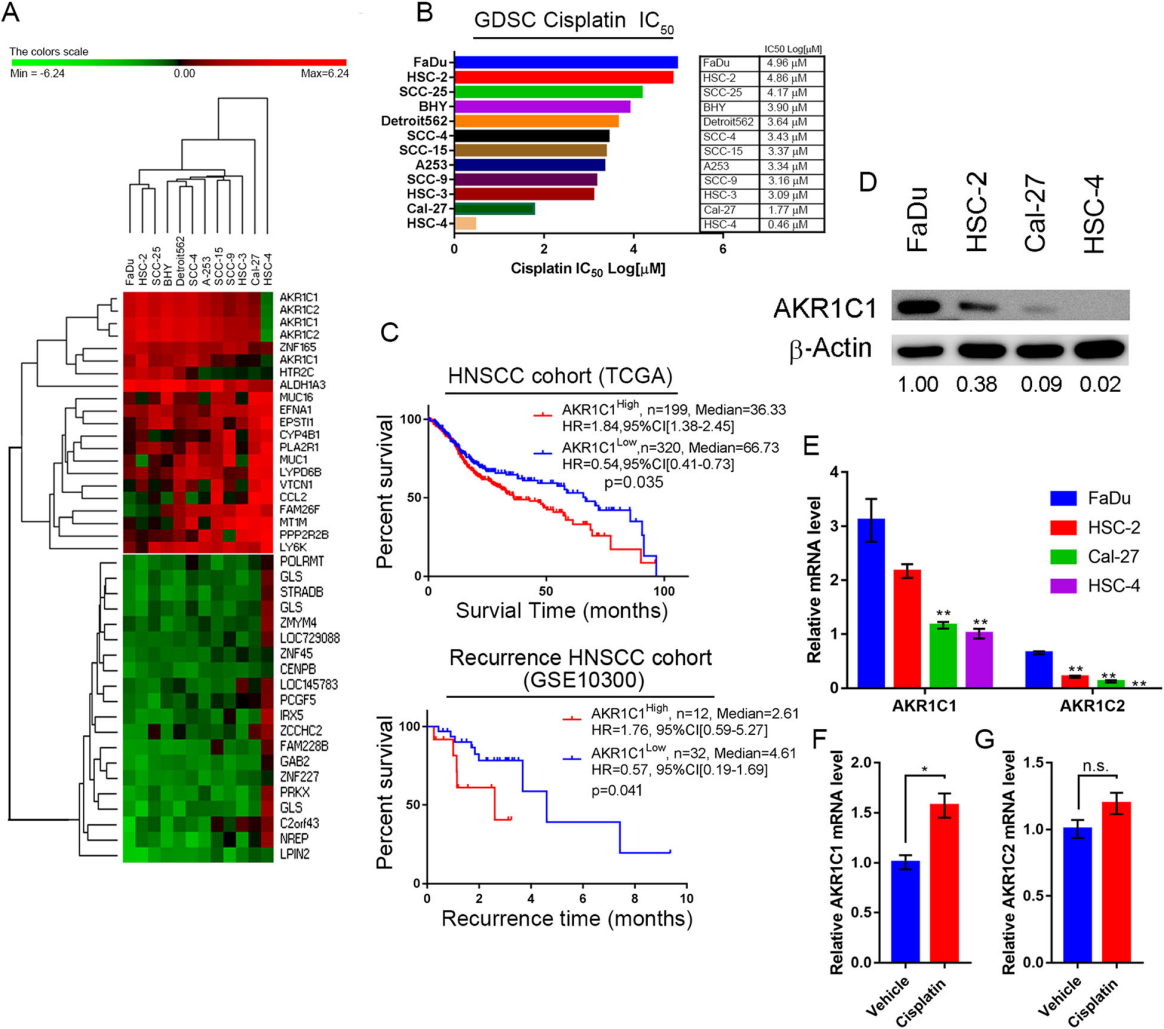
AKR1C1 expression correlates to cisplatin response and functions as a poor prognostic marker in HNSCC. **a** Hierarchical clustering of differentially expressed genes between cisplatin IC_50_ from GDSC and gene expression profiles from CCLE. Note that AKR1C1 and C2 are highly correlated with cisplatin IC_50_. **b** HNSCC cisplatin IC_50_ value from the GDSC database. **c** AKR1C1 expression was correlated with a poor survival rate in HNSCC patients in the TCGA (Upper panel, *n* = 519, HR = 1.84, *p* = 0.035) and a short recurrence-free time from the GSE10300 HNSCC cohort (Bottom panel, *n* = 44, HR = 1.76, *p* = 0.041) from the SurvExpress database. **d** and **e**. Expressions of AKR1C1 were assessed by immunoblotting (**d**) and RT-Q-PCR (**e**) in the indicated cell lines. **f** and **g**. The AKR1C1 (**f**) andAKR1C2 (**g**) mRNA expression level in Cal-27 cells with or without cisplatin treatment. The statistical significance was analyzed by Student’s t-test. **p < 0.01, ***p < 0.001

To find the genes correlated to cisplatin response in HNSCC cells, we combined the CCLE gene expression profiles (Fig. 1a) and cisplatin IC_50_ results from GDSC (Fig. 1b). We used PermutMatrix [21] to analyze the hierarchical clustering of HNSCC gene expression profiles based on cisplatin IC_50_ from GDSC. Intriguingly, most HNSCC cells were clustered together, except Cal-27 and HSC-4. AKR1C1 and AKR1C2 expression were downregulated in Cal-27 and HSC-4 cells. The multiple probes of AKR1C1/C2 which target to different sequence of AKR1C1/C2 mRNA and their consistency trends in the heat maps were correlated to cisplatin IC_50_. This observation indicated that AKR1C1/2 level might contribute to cisplatin response in HNSCC cells. However, the redundant enzymatic function and highly similar protein-coding sequences of AKR1C1 and AKR1C2 made them difficult to distinguish with commercial antibodies and functional assays. AKR1C1 and C2 may have the same gene regulation or cisplatin-resistance mechanisms in HNSCC cells. Thus, we chose AKR1C1 as an example to examine its role in cisplatin-resistance in HNSCC. We further analyzed the prognostic value and clinical characteristics information of AKR1C1 in HNSCC patients (Fig. 1c and Additional file 2: Table S2). High AKR1C1 expression level could be a poor prognostic marker in TCGA HNSCC cohort (hazard ratio, HR = 1.84, *p* = 0.035). Patients with higher AKR1C1 expression demonstrated shorter median survival time (36.33 months) than those with lower AKR1C1 expression (66.73 months). AKR1C1 is correlated to HPV p16 expression, lymph node metastasis by hematoxylin and eosin stain, SCC histologic grade and smoking history of the patient (Additional file 2: Table S2). Furthermore, in the recurrent HNSCC patient cohort (GSE10300), patients with higher AKR1C1 expression were prone to recur earlier (2.61 months) than those patients with lower AKR1C1 expression (4.61 months). We also examined the in vivo tumorigenesis ability (Additional file 3: Figure S1) and AKR1C1 expression level (Fig. 1d and e) in HNSCC cells. We found AKR1C1 was a more dominant form than AKR1C2 in HNSCC cells (Fig. 1e). Furthermore, we also found cisplatin treatment could significantly stimulate AKR1C1 mRNA expression but not AKR1C2 mRNA (Fig. 1f and g). Taken together, these results indicated that AKR1C1 expression could be a poor prognostic and recurrent biomarker in HNSCC patients.

### Silencing of AKR1C1 can increase cisplatin response in HNSCC through enzyme independent function

**Fig. 2:**
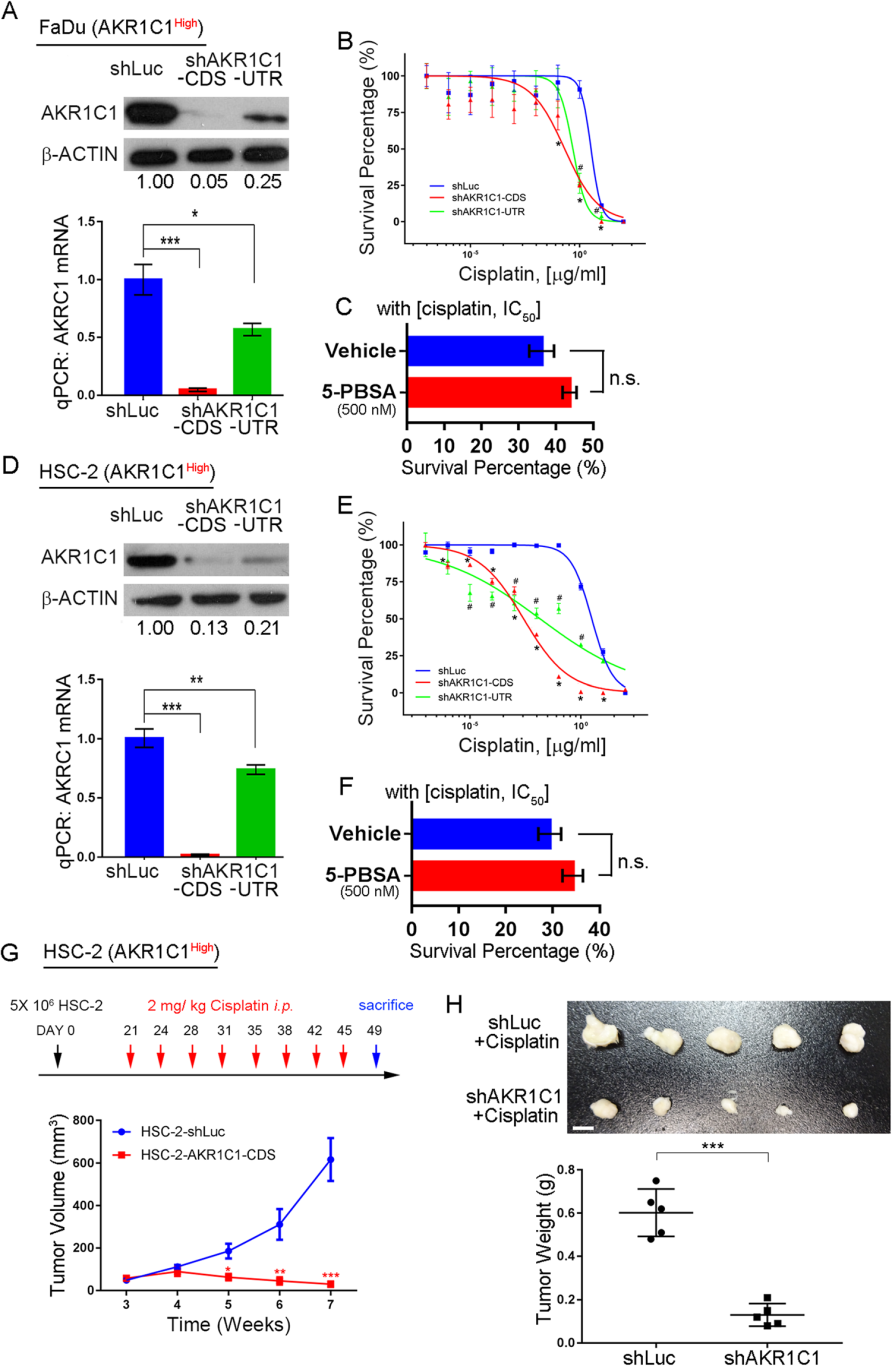
Silencing of AKR1C1 can increase the cisplatin response activity in HNSCC cells through enzyme-independent functioning. **a** to **f** The in vitro cell viability assay after combining cisplatin and shAKR1C1 lentiviral particles or enzymatic AKR1C1 inhibitor, 5-PBSA, in highly AKR1C1 expressed cells. **a** and **d** AKR1C1 protein (upper) and mRNA (bottom) expression after knockdown of AKR1C1. **b** and **e** Dose-response curve after knockdown of AKR1C1. **c** and **f** The cell viability assay under cisplatin IC_50_ and with or without AKR1C1 inhibitor, 5-PBSA (500 nM). **g** and **h** The HSC-2 in vivo cisplatin response assay in which cells were infected with or without AKR1C1-CDS knockdown clones. **g** The cisplatin regimen (upper) and in vivo tumor burden (bottom, *n* = 5). The cisplatin was given 2 mg/kg through intraperitoneal injection (*i.p.*).**h** The tumor image and tumor weights from (**g**) and the scale bar indicates 0.5 cm length. The statistical significance was analyzed by Student’s t-test. **p < 0.01, ***p < 0.001

To understand the AKR1C1 function in HNSCC related cisplatin-resistance, we performed AKR1C1 shRNA silencing in high AKR1C1 expressing cells, FaDu and HSC-2 (Fig. 2a and d). The shRNA clone that was targeted to the protein coding sequence (CDS) revealed higher efficacy than the clone targeted to the untranslated region (UTR). We used the curve-fitting model to indicate cisplatin response in FaDu and HSC-2 cells where the AKR1C1 expression was silenced by shAKR1C1 pseudoviruses. The IC_50_ values of the shAKR1C1 RNAs in FaDu cells when targeted to CDS were 686.55 nM with 95% CI = 382.27 to 1232.79 nM; when targeted to UTR were 1635.39 nM with 95% CI = 1182.13 to 2262.28 nM; and for the control shLuc were 11,061.45 nM with 95% CI = 7572.04 to 16,153.92 nM. In addition, the IC_50_ values of the shAKR1C1 RNAs in HSC-2 cells when targeted to CDS IC_50_ were 8.70 nM with 95% CI = 6.33 to 11.96 nM; when targeted to UTR were 47.53 nM with 95% CI = 16.86 to 133.91 nM; and for the control shLuc were 10,294.92 nM with 95% CI = 8808.50 to 12,031.29 nM, (Fig. 2b and e). The AKR1C1 expression level showed a comparable response of cisplatin toxicity in FaDu and HSC-2 cells. When FaDu and HSC-2 cells were cotreated with cisplatin with or without the AKR1C1 inhibitor, 5-PBSA [22], the cell viability was unchanged in both cells (Fig. 2c and f). These results indicated that the cisplatin-resistance function may not depend on AKR1C1 enzyme function. We further validated that in vivo AKR1C1 induced cisplatin-resistance in NSG mice (Fig. 2g). Reducing AKR1C1 expression in HSC-2 shAKR1C1 silenced cells could increase the cisplatin response effect compared with the shLuc control cells (Fig. 2h). These results indicated that AKR1C1 can promote cisplatin-resistance in an enzyme-independent manner.

### Ectopic AKR1C1 can promote cisplatin-resistance, anti-apoptosis response and cancer stemness in HNSCC

**Fig. 3:**
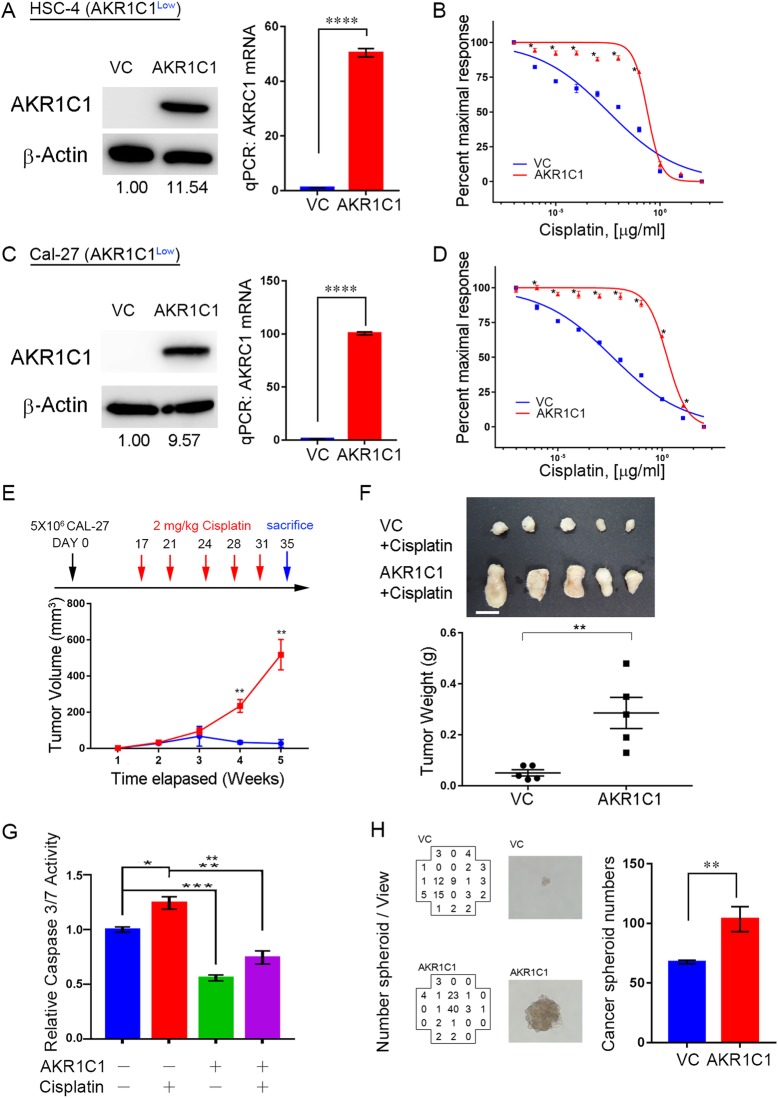
Ectopic AKR1C1 can promote cisplatin-resistance, anti-apoptosis response and cancer stemness in HNSCC cells. **a** to **d** The in vitro cell viability assay after combining cisplatin and ectopic AKR1C1 lentiviral particles. **a** and **c** AKR1C1 protein (left) and mRNA (right) expression after enforced expression of AKR1C1. **b** and **d** Dose-response curve after enforced expression of AKR1C1. **e** and **f** The Cal-27 in vivo cisplatin response assay in which cells were infected with or without AKR1C1 overexpression clones. **e** The cisplatin regimen (upper) and in vivo tumor burden (bottom, *n* = 5). The cisplatin was given 2 mg /kg through intraperitoneal injection (*i.p.*). **f** The tumor image and tumor weights from (**e**) and the scale bar indicates 1 cm length. **g** The cisplatin-induced caspase 3/7 activity assay with or without AKR1C1 expression. **h** The cancer spheroid formation assays with or without AKR1C1 expression in Cal-27 cells. The left panels indicate spheroid numbers which were calculated by ImageXpress XLS High-content system and the middle panels indicate the representative spheroid image in high magnification. The statistical significance was analyzed by Student’s t-test. **p < 0.01, ***p < 0.001

**Fig. 4:**
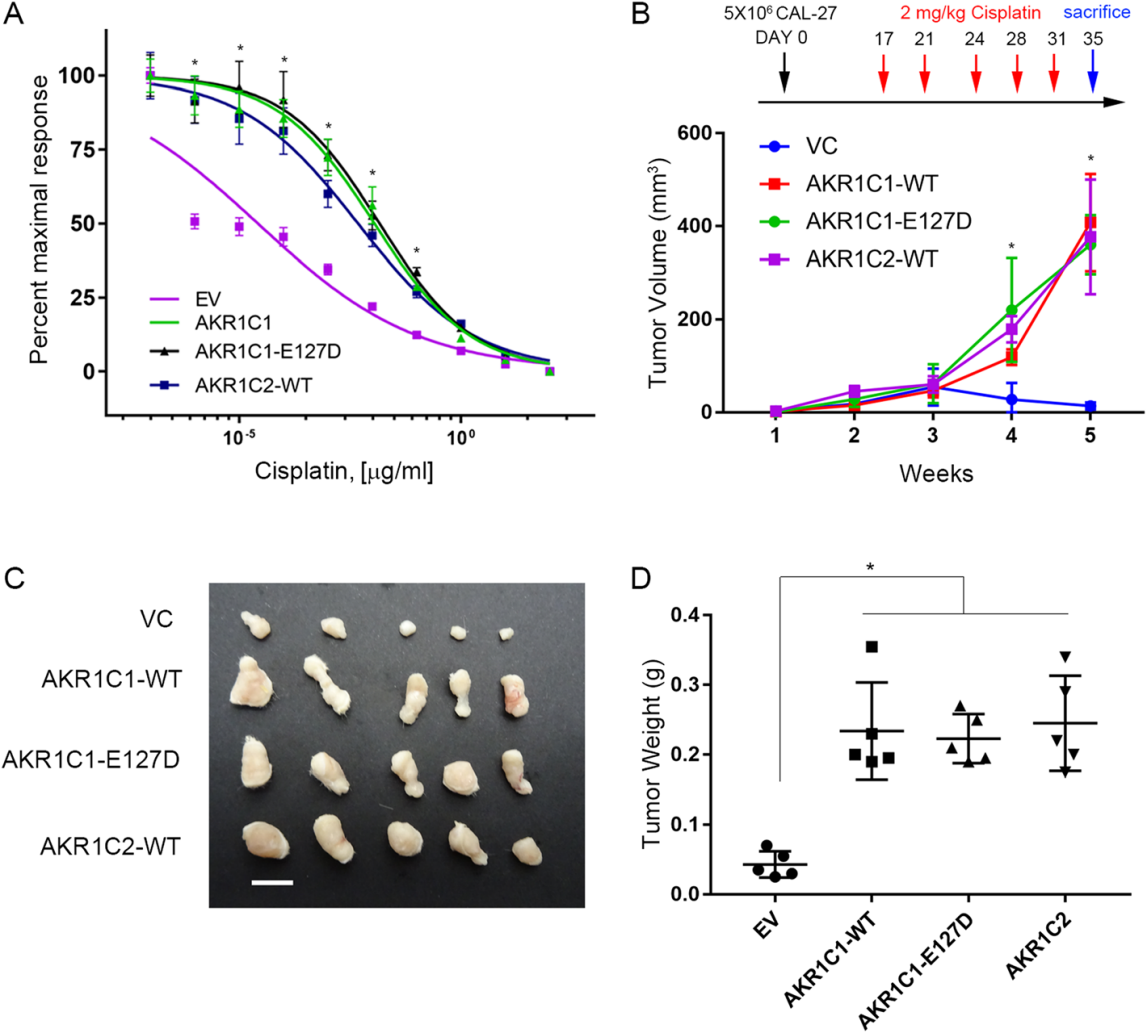
AKR1C1 promotes cisplatin-resistance enzymatic-independent manner. **a** Cisplatin dose-response curve in wild type AKR1C1, AKR1C2, and domain-negative AKR1C1-E127D Cal-27 cells. **b** to **d**. **b** The cisplatin regimen (upper) and in vivo tumor burden (bottom, *n* = 5). The cisplatin was given 2 mg /kg through intraperitoneal injection (*i.p.*). **c** and **d**. The tumor image and tumor weights (**d**) from (**c**) and the scale bar indicates 1 cm length

To further investigate the role of AKR1C1 in cisplatin-resistance, we used the two-way model to analyze AKR1C1 functions in cells expressing lower, endogenous levels of AKR1C1, HSC-4 and Cal-27 (Fig. 3a and c). Ectopic AKR1C1 expression could increase the cisplatin IC_50_ from 11.28 nM (Fig. 3b, HSC-4, 95% CI = 7.085 to 17.96 nM) to 833.19 nM (95% CI = 685.55 to 1012.83 nM), and 14.34 nM (Fig. 3d, Cal-27, 95% CI = 10.72 to 19.20 nM) to 5805.68 nM (95% CI = 10,717.18 to 19,196.73 nM), respectively. Cal-27 cells demonstrated higher in vivo tumorigenesis ability in NSG mice than HSC-4 cells (Additional file 3: Figure S1); thus, we chose Cal-27 for further in vivo cisplatin response assays in NSG mice and in vitro mechanism assays of cisplatin-resistance. Compared to empty vector control (VC), AKR1C1 expression could decrease the cytotoxicity effects in Cal-27 cells (Fig. 3e and f). Furthermore, AKR1C1 expression could reduce the caspase activity in cisplatin-treated Cal-27 cells (Fig. 3g). Cancer stem cells play a major role in drug resistance and tumor recurrence and AKR1C1/C2 are upregulated in a minor population of lung cancer stem cells [23]. We further examined the cancer sphere formation ability of AKR1C1, which could increase the cancer spheroids in Cal-27 cells (Fig. 3h). In order to prove that AKR1C1 induced cisplatin-resistance through enzymatic-independent manner, we used domain negative AKR1C1-E127D clone and found that both wild type AKR1C1 and AKR1C1-E127D clone could induce cisplatin resistance in Cal-27 (Fig. 4a to d). These results indicated that AKR1C1 expression could increase cisplatin-resistance in HNSCC.

### AKR1C1 induces STAT activation and influences downstream survival and inflammatory signaling in HNSCC

**Fig. 5:**
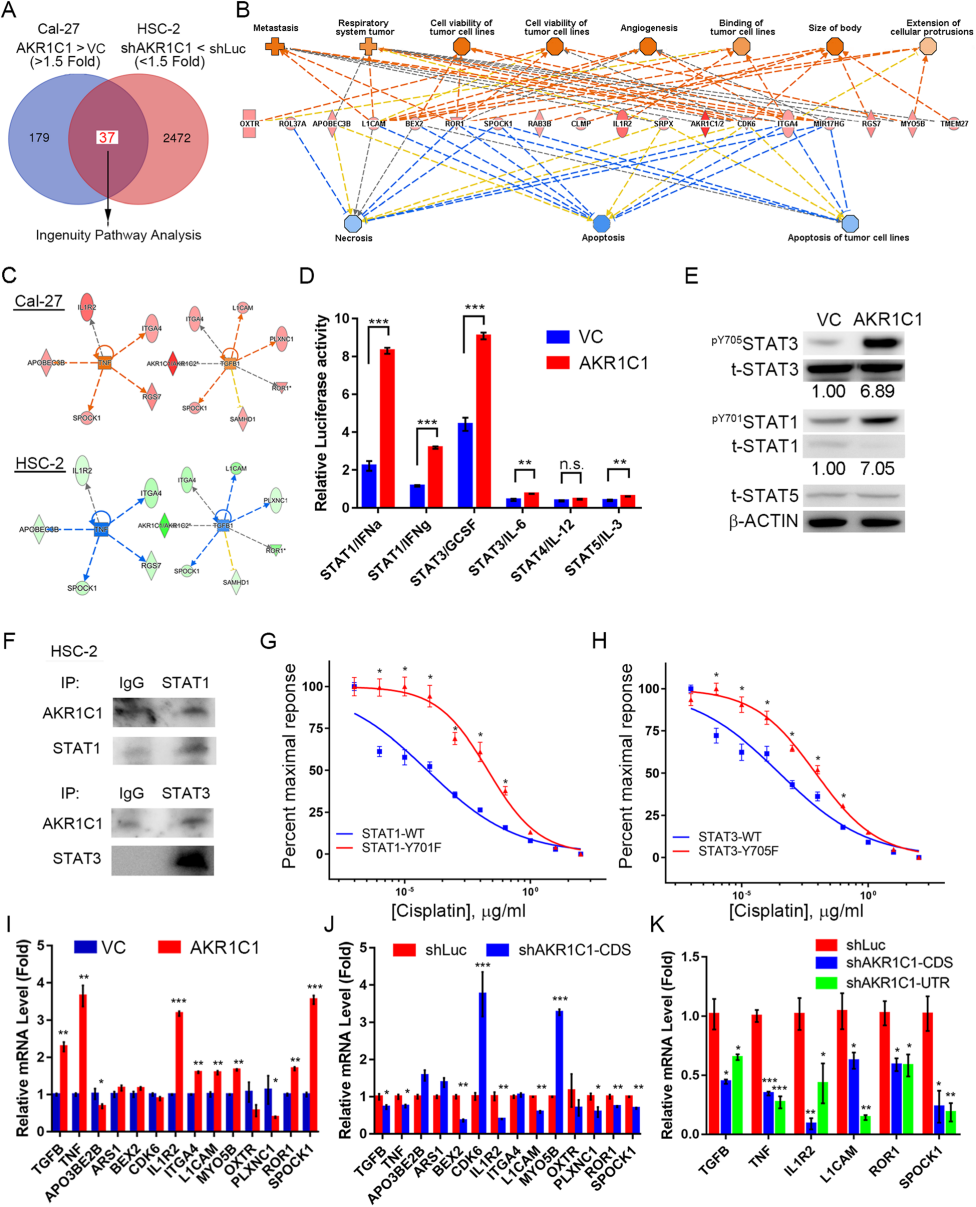
AKR1C1 controls anti-cell death pathways and inflammatory gene networks in HNSCC cells. **a** The flowchart of identifying the AKR1C1 downstream genes with 1.5-fold change cutoff compared to control vectors and their possible regulators in HNSCC cells. **b** The disease and biological function results from the IPA database **c** AKR1C1 regulates inflammation proteins, such as TNF-α and TGF-β networks, by IPA in HSC-2 (**d**) and Cal-27 (**e**) cells. D. STAT family reporter activity assays. The reporter activity was normalized by the expression level of pGL4-miniP reporters in the stable cell lines. **e** Phosphorylation level of ^pY705^STAT3 and ^pY701^STAT1 in AKR1C1-expressing Cal-27 cells. **f** Immunoprecipitation STAT1 and STAT3 in HSC-2 cells. **g** and **h** Cisplatin dose-response curve in Cal-27 expression with wild type STAT1 or constitutive activation form STAT1-Y701F (**g**) or STAT3 or STAT3-Y705F. I to **k**. The real-time PCR validation of microarray candidates in AKR1C1 overexpression (I, Cal-27) and knockdown (J, HSC-2; K, FaDu) cells. The gene expressions were normalized with endogenous GAPDH expression. The statistical significance was analyzed by Student’s t-test. *p < 0.05 **p < 0.01, ***p < 0.001

To ascertain the mechanism by which AKR1C1 induced cisplatin-resistance in HNSCC, we performed microarray analysis in a two-way model of AKR1C1 overexpression and knockdown in Cal-27 and HSC-2 cells (Fig. 5a). The normalized data from the microarray analysis were subjected to IPA to identify the crosstalk of the AKR1C1 signaling network in HNSCC (Additional file 4: Table S3 and Additional file 5: Table S4). AKR1C1 expression activated several oncogenic functions including tumor cell viability, metastasis, and angiogenesis. Several tumor suppression pathways including apoptosis and necrosis were suppressed by AKR1C1 expression (Fig. 5b and Additional file 4: Table S3). Taken together, these results suggest that AKR1C1 might contribute to tumor progression and prevent tumor cell death in HNSCC. Moreover, we also analyzed the dynamics of cellular regulators upon AKR1C1 expression, finding that the downstream genes belonging to TNF and TGFB1 were controlled by AKR1C1. (Fig. 4c and Additional file 5: Table S4). Interestingly, both downstream regulators belonged to inflammatory genes and were also controlled by oncogenic signaling proteins, signal transducer and activator of transcription (STAT) families, in different cell types. Recently, Zhu et al. reported an AKR1C1 enzymatic-independent function in STAT3 activation in lung cancer cells [24]. Thus, we used the STAT family luciferase reporters to examine the STAT activity under AKR1C1 expression. In Cal-27 cells, AKR1C1 expression promoted STAT1 and STAT3 transcriptional activity and stimulated protein phosphorylation in transcriptional activation and dimerization tyrosine residues Y705 and Y701 in STAT3 and STAT1, respectively (Fig. 5d and e). STAT1 and STAT3 had direct interaction with AKR1C1 in HSC-2 cells which had high cisplatin IC_50_ and AKR1C1 expression (Fig. 5f). Moreover, the constitutive activation STAT1-Y701F and STAT3-STAT3 could promote cisplatin-resistance in Cal-27 cells (Fig. 5e and f). The tyrosine phosphorylation of STAT1 and STAT3 are catalyzed by Janus kinase (JAK) and control the STAT1 and 3 downstream transcriptome, including the TGF-B1 and TNF gene network, and promote tumor progression and chemoresistance in cancer cells [25, 26]. In HNSCC cells, we used real-time quantitative PCR to validation the microarray and IPA analysis upon AKR1C1. AKR1C1 controlled TGFB1, TNF gene expressions and several downstream genes including IL1R2, L1CAM, ROR1 and SPOCK1 in HNSCC cells (Fig. 5i to k). These results indicated that AKR1C1 promotes the oncogenic signaling STAT1 and STAT3 activation and downstream signaling that might contribute to cisplatin-resistance in HNSCC.

### Cigarette metabolites promote AKR1C1 expression and STAT1 and 3 activation in HNSCC

**Fig. 6:**
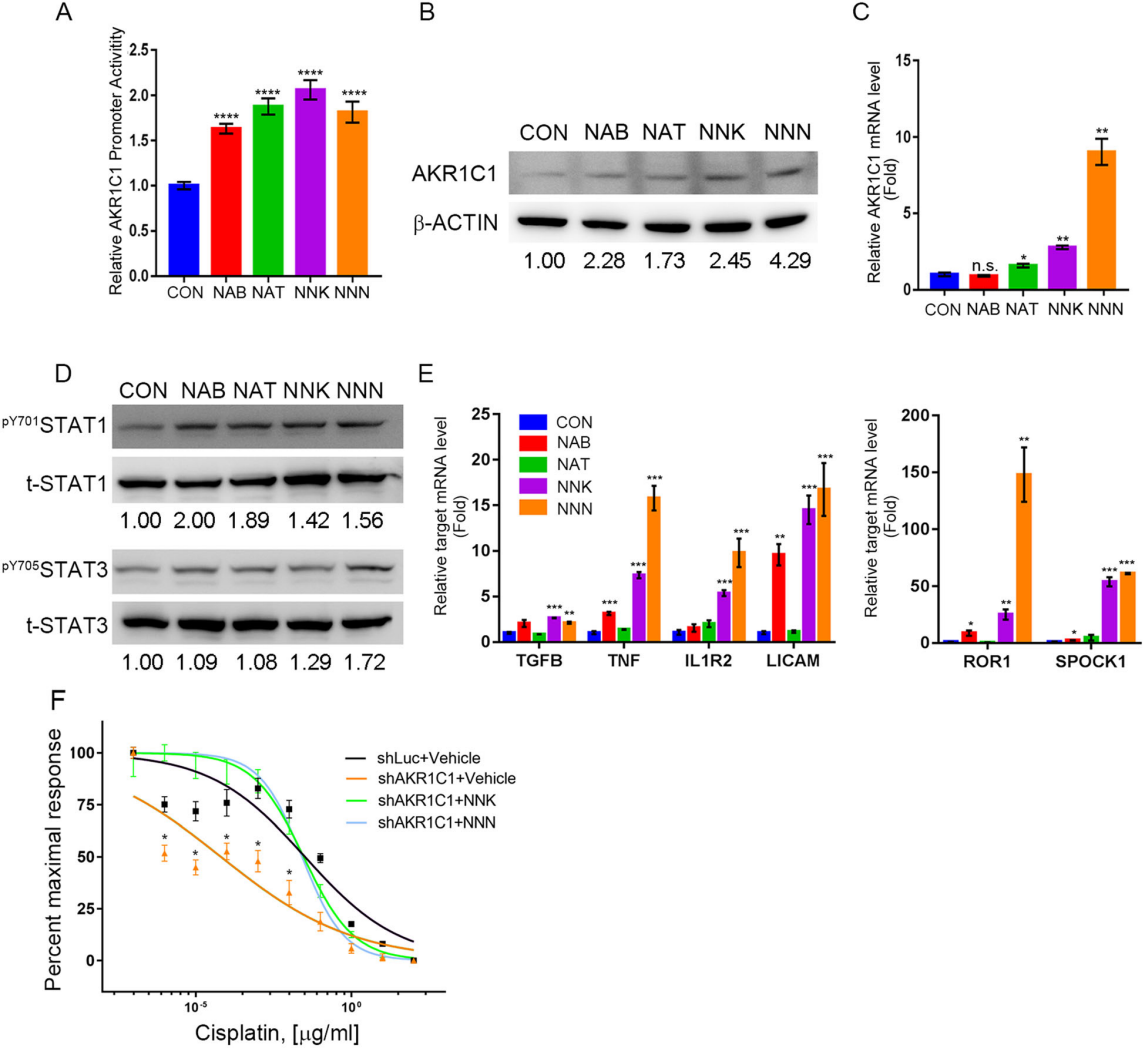
Cigarette metabolites control AKR1C1 expression. **a** to **c** AKR1C1 promoter activity assay (**a**), protein (**b**) and mRNA level (**c**) in Cal-27 cells exposed to tobacco-specific nitrosamines (TSNA), such as NAB, NAT, NNK and NNN and vehicle at concentration (10 μM) for 24 h. **d** The phosphorylation status of ^pY701^STAT1 and ^Py705^STAT3 in Cal-27 cells exposed to TSNA. **e** The AKR1C1 induced STAT1 and 3 downstream gene expression in Cal-27 exposed to TSNA. The gene expressions were normalized by endogenous GAPDH expression. **f** Cisplatin dose-response curve after knockdown of AKR1C1 and co-treat with NNK or NNN. The statistical significance was analyzed by Student’s t-test. *p < 0.05 **p < 0.01, ***p < 0.001

AKR1C1 is highly correlated to patient smoking history (Additional file 2: Table S2) and is also the most important detoxification enzyme of NNK (17). NNK and other tobacco-specific nitrosamines (TSNA), including NAB, NAT and NNN, are major carcinogens in cigarette smoking, which directly exposes the oral cavity and airway. In NSCLC cells, NNK treatment stimulates proliferation and inhibits chemotherapy-induced apoptosis through AKT and NF-kB activation [27, 28]. Thus, we examined whether exposure to TSNAs induces AKR1C1 and downstream gene expression in HNSCC cells. All TSNA exposure stimulated AKR1C1 promoter activity and AKR1C1 protein and mRNA expression in Cal-27 cells (Fig. 6 a-c). Using microarray analysis, we found TSNAs treatment could active the STAT3 signature in Cal-27 cells (Additional file 6: Table S5). Thus, we examined the phosphorylation statuses of STAT1 and 3 and their downstream gene expressions under TSNAs exposure (Fig. 6 d & e). Furthermore, NNK and NNN could abolish the cisplatin toxicity in AKR1C1 knockdown cells (Fig. 6f). NNK and NNN had stronger effects than NAB and NAT in AKR1C1 induction and STAT signaling activation in Cal-27 cells. These results suggest that overexpression of AKR1C1 in HNSCC may be induced by cigarette smoking.

### JAK inhibitor, ruxolitinib, prevents AKR1C1 induced JAK-STAT signaling and cisplatin-resistance

**Fig. 7:**
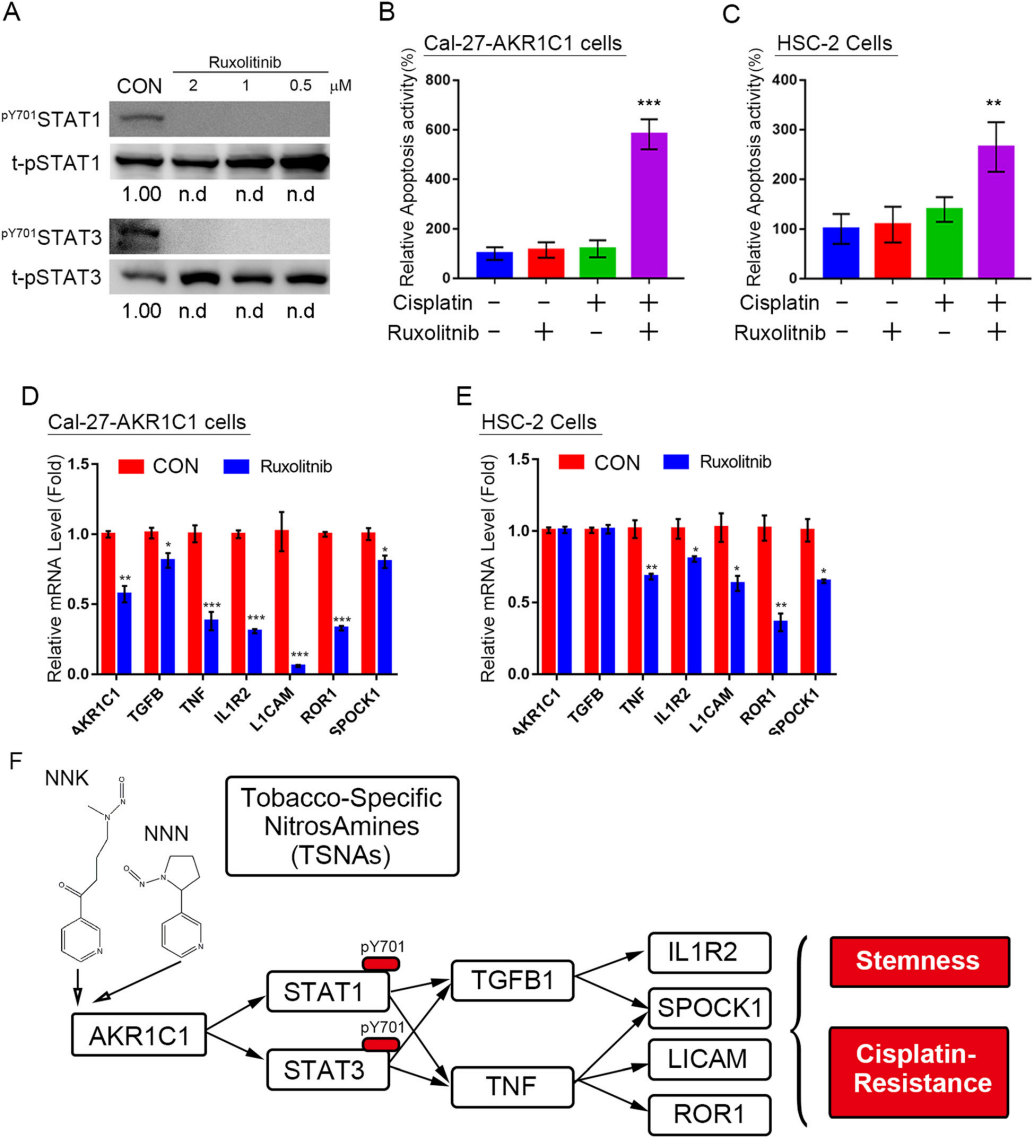
The JAK inhibitor ruxolitinib inhibits AKR1C1-induced cisplatin-resistance and JAK-STAT signaling pathway activation. **a** immunoblotting of phosphorylation ^pY701^STAT1 and ^pY705^STAT3 status under ruxolitinib-treated Cal-27 AKR1C1 cells (2–0.5 μM). **b** and **c** The caspase 3/7 activity assay in Cal-27-AKR1C1 (**b**) and HSC-2 (**c**) cells. The cisplatin concentration is 5 μM in Cal-27 cells and 10 μM in HSC-2 cells. The ruxolitinib is treated 0.5 μM in both cells. **d** and **e** The real-time PCR results of STAT3 downstream gene expression in Cal-27-AKR1C1 (**d**) and HSC-2 (**e**) cells. **f** The hypothetical model of the AKR1C1 contribution to cisplatin-resistance through STAT1 and 3 activation in HNSCC. The gene expressions were normalized with endogenous GAPDH expression. The statistical significance was analyzed by Student’s t-test. n.d: non-detected, *p < 0.05 **p < 0.01, ***p < 0.001

Ruxolitinib is a FDA-approved oral JAK inhibitor which has been used to treat patients with myeloproliferative disorders or polycythemia vera [29, 30]. Ruxolitinib is also used to treat immunodysregulation in patients with gain of function STAT1 or STAT3 mutations [31]. Recently, ruxolitinib has been tested in many clinical trials in different cancer types, including a phase 2 trial in head and neck cancer [32]. Thus, we examined the cisplatin sensitivity role of ruxolitinib in overcoming AKR1C1-induced cisplatin-resistance. In Cal-27 cells, ruxolitinib treatment could prevent STAT1^Y701^ and STAT3^-Y705^ phosphorylation, both of which were contributed by AKR1C1 (Fig. 7a). Combining ruxolitinib and cisplatin increased the apoptotic cell ratio in both Cal-27-AKR1C1 cells (Fig. 7b) and HSC-2 cells (Fig. 7c). Moreover, the expression level of AKR1C1-induced STAT1 and 3 target genes were also suppressed by ruxolitinib treatment in both cell types (Fig. 7d and e). Taken together, the results indicate that ruxolitinib might contribute to overcoming cisplatin-resistance in HNSCC.

## Discussion

In this study, we determined that AKR1C1 may account for cisplatin-resistance via activating STAT signaling pathways, ultimately resulting in poor clinical outcome. Cisplatin has been the standard chemotherapy for most upper aerodigestive tract carcinomas, including HNSCC, lung cancer, and esophageal cancer, for decades. Mechanisms of cisplatin-resistance have also been discussed, and currently there are several major factors that are considered to contribute to it: membrane transporters for cisplatin uptake or efflux, such as CRT1 and ABC transporter MRP2; DNA repair proteins, such as ERCC1 and TP53; apoptosis associated proteins including BCL-2, caspases, or MAPKs [5]. Since primary cisplatin-resistance has been regarded as a very poor prognostic factor and many clinical trials have excluded patients who recurred within 6 months after primary or adjuvant cisplatin-based chemoradiotherapy in HNSCC, understanding additional details in cisplatin-resistance mechanisms can enable clinical oncologists and medical researchers to design novel therapeutic strategies for these cancer patients.

AKR1C1 expression is a poor prognostic marker in a wide variety of cancers, including breast, prostate, non-small cell lung, and esophagus [33], and it is upregulated in recurrent tumors and cancer stem cells [23, 33]. In acquired cisplatin-resistance and metastatic ovarian and gastric cancer cells, AKR1C1 is upregulated by IL-6 stimulation and nuclear factor erythroid 2-related factor 2 (Nrf2) and promotes chemoresistance. [34–38]. Because AKR1C1 works as cellular ROS scavenger and up-regulation in cancer-stem cells which hint that AKR1C1 might be a broad-range chemoresistance gene in cancer. However, the molecular mechanism remains unclear of AKR1C1 in HNSCC cisplatin-resistant. In this study, we found AKR1C1 contributing to cisplatin-resistance and cancer stemness phenotype in HNSCC. Furthermore, we found that cigarette metabolites could induce AKR1C1 expression and further activate STAT signaling. Previously, we identified an AKR1C1 enzymatic-independent mechanism that induced STAT1 and STAT3 activation in treatment-naïve NSCLC cells [24] and demonstrated that this activation could be attenuated by ruxolitinib. STATs are key regulators which stimulate IL-6 expression. AKR1C1, STATs, and cytokine IL-6 potentially form a positive feedback loop in cancer to enhance cisplatin-resistance.

Smoking is one of the most important environmental carcinogens that induces “field cancerization” in upper aerodigestive tract carcinoma [39]. There are more than 20 carcinogens involved in tobacco [40], and the smaller particles, such as air pollutants from second-hand smoke, may predominantly deposit in lung parenchyma and promote local inflammation. TSNAs, specifically NNK, promote NSCLC proliferation and prevent chemotherapy-induced apoptosis (32, 33); however, studies to provide similar information for HNSCC are relatively few. In this study, we determined that the similar chemical structure of NNK and NNN lead to more potent STAT-stimulating activity than NAB and NAT. These phenomena may arise from the inhibitory function of NNK in E3-ligase protein and βTrCP [41] and prevent degradation of EMI1 and CTNNB1 [42] . The situation found in the oral cavity and upper aerodigestive tract may also occur in the lung. From this study we observed that AKR1C1 was overexpressed after exposure to TSNAs, resulting in STAT activation and cisplatin-resistance (Fig. 7f). The reason why TSNAs would induce AKR1C1 expression may be due to the compensatory metabolic effects of the cells. Based on this evidence, upper aerodigestive tract carcinoma patients who are receiving cisplatin treatment should cease smoking immediately to prevent acquired cisplatin-resistance.

Ruxolitinib is a JAK1/2 inhibitor that targets STAT-associated signaling. It has been approved by the FDA for hematologic premalignancy including myelofibrosis and polycythemia vera. Clinical studies of ruxolitinib primarily focus on hematologic malignancies and some aggressive solid cancers that may harbor stem-like features, such as glioblastoma multiforme or triple negative breast cancer. In HNSCC, STAT3 activation has been observed in tumors and may be regulated by upstream EGFR overexpression, IL-6 inflammatory cytokines, or additional pathways. Targeting STAT3 to overcome drug resistance in HNSCC has been discussed, but trials are seldom conducted using ruxolitinib [43]. In this study, we showed that ruxolitinib could overcome intrinsic cisplatin-resistance in HNSCC. Further studies focusing on targeting this novel AKR1C1/STAT network are warranted.

## Conclusions

This is the first conceptual link between cigarette metabolites which induce AKR1C1 overexpression and cisplatin resistance in HNSCC and cause poor prognosis. The AKR1C1/STAT crosstalk is associated with primary cisplatin-resistance and may be overcome by the JAK inhibitor ruxolitinib. Whether AKR1C1 could be an effective prognostic or predictive factor for HNSCC patients treated with cisplatin-based chemotherapy warrants further validation. New combination therapy with cisplatin and drugs targeting AKR1C1/STAT signaling also may be beneficial to primary cisplatin-resistant HNSCC patients.

## Additional files

Additional file 1: **Table S1.** Reagents and primer information in this manuscript (DOCX 23 kb)

Additional file 2: **Table S2.** Clinical Characteristics of AKR1C1 in TCGA HNSCC cohorts (DOCX 24 kb)

Additional file 3: **Figure S1.** In vivo tumor growth abilities in HNSCC cells. **Figure S2**. Full length Western blot images. (DOCX 2354 kb)

Additional file 4 **Table S3.** AKR1C1 controls cellular functions from Ingenuity Pathway Analysis (DOCX 17 kb)

Additional file 5 **Table S4.** AKR1C1 regulates Up-stream Regulators from Ingenuity Pathway Analysis (DOCX 16 kb)

Additional file 6 **Table S5.** The Up-stream Regulators of TSNAs treatment in Cal-27 cells from Ingenuity Pathway Analysis (DOCX 20 kb)

## Data Availability

The microarray data were uploaded to the National Center for Biotechnology Information Gene Expression Omnibus (GEO, NCBI, GSE119444). Chemical reagents, vectors, and antibodies are listed in Additional file 1: Table S1.

## Abbreviations

GEO: Gene Expression Omnibus
HNSCC: Head and neck squamous cell carcinoma
NAB: N′-nitrosoanabasine
NAT: N′-nitrosoanatabine
NNK: 4-(methylnitrosamino)1-(3-pyridyl)-1-butanone
NNN: N-nitrosonornicotine
NSG: Nod-SCID gamma
TSNAs: Tobacco-specific nitrosamines
