# Supplementary material for: AKR1C1 controls cisplatin-resistance in head and neck squamous cell carcinoma through cross-talk with the STAT1/3 signaling pathway
Source: J Exp Clin Cancer Res. 2019 Jun 10;38:245. doi: 10.1186/s13046-019-1256-2 (PMC6558898; doi:10.1186/s13046-019-1256-2)
Supplement: Supplementary file 1 — Table S1. Reagents and primer information in this manuscript (DOCX 23 kb) [file 13046_2019_1256_MOESM1_ESM.docx]

| **Table S1: Reagents and primer information in this manuscripts** | | | |  | | |
| --- | --- | --- | --- | --- | --- | --- |
|  |  | | |  | |  |
| Chemical reagents |  | | |  | |  |
| Name | Brand | | | Catalogue number | |  |
| Cisplatin | Sigma-Aldrich | | | P4394 | |  |
| 5-PBSA | Cayman | | | 13574-1MG | |  |
| Hoechst 33342 | Invitrogen | | | H3570 | |  |
| Alamar blue | Sigma-Aldrich | | | R7017 | |  |
| ONE-Glo™ Luciferase Assay System | Promega | | | E6110 | |  |
| Human Genomic DNA | Clontech | | | 636401 | |  |
| HE Swift Cloning Kit | Biotools Co. | | | TB-VTT-BB05 | |  |
| OmicsGreen qPCR MasterMix. | Omics Bio | | | QE3933 | |  |
| LR Clonase II Plus enzyme | Invitrogen | | | 12538-200 | |  |
| Ruxolitinib | Incyte Corporation | | |  | |  |
| (R,S)-N-Nitrosoanabasine (NAB) | Toronto Research Chemicals | | | N524250 | |  |
| (S)-N-Nitroso Anatabine (NAT) | Toronto Research Chemicals | | | N524745 | |  |
| 4-(Methylnitrosoamino)-1-(3-pyridinyl)-1-butanone (NNK) | Sigma-Aldrich | | | FL-78013 | |  |
| N-Nitrosonornicotine (NNN) | Sigma-Aldrich | | | FL-75285 | |  |
|  |  | | |  | |  |
| Vector |  | | |  | |  |
| Name | Brand | | | Catalogue number | |  |
| pCT-Apoptosis-Luc | SBI | | | CYTO114-PA-1 | |  |
| pLenti6.3-DEST | Invitrogen | | | V53306 | |  |
| pDONR221-AKR1C1 | DNASU | | | HsCD00043501 | |  |
| pGL4[Luc2P/GCSF/Hygro] | Promega | | | CS181505 | |  |
| pGL4.45[luc2P/ISRE/Hygro] | Promega | | | E4141 | |  |
| pGL4[Luc2P/GAS-RE/Hygro] | Promega | | | CS179301 | |  |
| pGL4.47[luc2P/SIE/Hygro] | Promega | | | E4041 | |  |
| pGL4[Luc2P/STAT4-RE/Hygro] | Promega | | | CS181501 | |  |
| pGL4.52[luc2P/STAT5/Hygro] | Promega | | | E4651 | |  |
| pGL4.26[luc2/minP/Hygro] | Promega | | | E8441 | |  |
| pGreenFire1-mCMV (EF1α-puro) | SBI | | | TR010PA-P | |  |
|  |  | | |  | |  |
| shRNA sequence |  | | |  | |  |
| Name | Brand | | | Clone ID | | Target Seqence |
| pLKO-shLuc | RNAiCore | | | TRCN0000072249 | | GCGGTTGCCAAGAGGTTCCAT |
| pLKO-shAKR1C1-CDS | RNAiCore | | | TRCN0000344631 | | ATGTTGACCTCTACCTTATTC |
| pLKO-shAKR1C1-UTR | RNAiCore | | | TRCN0000344685 | | GACACAGAGGATGGCTCTATG |
|  |  | | |  | |  |
|  |  | | |  | |  |
| Antibodies |  | | |  | |  |
| Name | Brand | | Catalogue number | dilution | | Dilution |
| AKR1C1 | Genetex | | GTX105620 | 1:1000 | | 1:1000 |
| beta-Actin | Sigma | | HPA041271 | 1:5000 | | 1:5000 |
| phosphoSTAT1-Y701 | Cell signaling | | #8826 | 1:1000 | | 1:1000 |
| phosphoSTAT3-Y707 | BOSTER | | P00007-2 | 1:1000 | | 1:1000 |
| STAT1 | Cell signaling | | #9172 | 1:1000 | | 1:1000 |
| STAT3 | Cell signaling | | #9139 | 1:1000 | | 1:1000 |
| STAT5 | Cell signaling | | #9363 | 1:1000 | | 1:1000 |
|  |  | | |  | |  |
| Primers |  | | |  | |  |
| Primer Name | Sequence | | |  | |  |
| AKR1C1-Pro1-R0-BamH1 | CAG GAT CCT GTC ACT AGC CTG GCT GGC AAA | | |  | |  |
| AKR1C1-Pro2-F-1276-EcoRI | CAG AAT TCC CCC TGG CCT TTT GCC TAT A | | |  | |  |
| AKR1C1-F210-223 | GCCATATTGATTCTGCTCATTTAT | | |  | |  |
| AKR1C1-R322-339 | TGGGAATTGCTCCAAAGC | | |  | |  |
| AKR1C2-F-210-233 | ACCATATTGATTCTGCACATGTTT | | |  | |  |
| AKR1C2-R-322-339 | TGGGAATTGCTCCAAAGC | | |  | |  |
| APOBEC3B-F-103-123 | ATCATCGGCAATAGCAGTGTG | | |  | |  |
| APOBEC3B-R-295-275 | AGGCTGTGGTGATAGTCCTGT | | |  | |  |
| BEX2-F-103-124 | AAAGAGGAACGAGCGTTAAACA | | |  | |  |
| BEX2-R-227-205 | TCACTAACATTCAAAGGTAGGGC | | |  | |  |
| OXTR-F-654-674 | CTGCTACGGCCTTATCAGCTT | | |  | |  |
| OXTR-R-895-877 | CGCTCCACATCTGCACGAA | | |  | |  |
| MYO5B-F-306-327 | CTGTGGTATCGTACTTGTTGCC | | |  | |  |
| MYO5B-R-489-467 | CCCACTGACTATGATGGACTGAT | | |  | |  |
| ROR1-F-167-188 | CTTACCTGACCCTCGATGAACC | | |  | |  |
| ROR1-R-338-317 | CCATAGATGGTGGACCGAAAGG | | |  | |  |
| L1CAM-F-105-125 | TGTCATCACGGAACAGTCTCC | | |  | |  |
| L1CAM-R-350-331 | CTGGCAAAGCAGCGGTAGAT | | |  | |  |
| SPOCK1-251-271 | ACCCCTGCCTGAAGGTAAAAT | | |  | |  |
| SPOCK1-413-393 | GGCTTGCACTTGACCAAATTC | | |  | |  |
| PLXNC1-F-1278-1300 | CTACAAACTCGTTCCTGATCCTG | | |  | |  |
| PLXNC1-R-1403-1383 | GTGGCTGTTAAACACTCCGAA | | |  | |  |
| IL1R2-F-395-415 | TCCTGCCGTTCATCTCATACC | | |  | |  |
| IL1R2-R-589-567 | CATCGTGTACGAGTAAGTGAGTG | | |  | |  |
| ITGA4-F-322-342 | AGCCCTAATGGAGAACCTTGT | | |  | |  |
| ITGA4-R-488-467 | CCAGTGGGGAGCTTATTTTCAT | | |  | |  |
| CDK6-F-25-44 | GCTGACCAGCAGTACGAATG | | |  | |  |
| CDK6-R-249-228 | GCACACATCAAACAACCTGACC | | |  | |  |
| TGFB1-F-334-355 | | CTAATGGTGGAAACCCACAACG | | |  | |
| TGFB1-R-542-522 | | TATCGCCAGGAATTGTTGCTG | | |  | |
| TNF-F-198-219 | | CCTCTCTCTAATCAGCCCTCTG | | |  | |
| TNF-R-417-397 | | GAGGACCTGGGAGTAGATGAG | | |  | |
| AKR1C1-E127D-S | | GTAAAGCCAGGTGAGGACGTGATCCCAAAAGATGAA | | |  | |
| AKR1C1-E127D-AS | | ATCTTTTGGGATCACGTCCTCACCTGGCTTTACAGA | | |  | |
