# Supplementary material for: AKR1C1 controls cisplatin-resistance in head and neck squamous cell carcinoma through cross-talk with the STAT1/3 signaling pathway
Source: J Exp Clin Cancer Res. 2019 Jun 10;38:245. doi: 10.1186/s13046-019-1256-2 (PMC6558898; doi:10.1186/s13046-019-1256-2)
Supplement: Supplementary file 2 — Table S2. Clinical Characteristics of AKR1C1 in TCGA HNSCC cohorts (DOCX 24 kb) [file 13046_2019_1256_MOESM2_ESM.docx]

| **Table S2: Clinical Characteristics of AKR1C1 in TCGA HNSCC cohorts** | | | | | | | |
| --- | --- | --- | --- | --- | --- | --- | --- |
|  |  | AKR1C1 |  |  |  |  |  |
|  |  | AKR1C1 Low | | AKR1C1 High | |  |  |
|  |  | n | % | n | % | n | p value |
| Gender | Female | 74 | 55.22% | 60 | 44.78% | 508 |  |
|  | Male | 180 | 48.13% | 194 | 51.87% |  |  |
| Age at diagnosis | <= 65y | 163 | 51.10% | 156 | 48.90% | 508 |  |
|  | > 66y | 91 | 48.15% | 98 | 51.85% |  |  |
| Primary site | lower risk | 53 | 56.38% | 41 | 43.62% | 508 |  |
|  | higher risk | 201 | 48.55% | 213 | 51.45% |  |  |
| Clinical T Status | T1+T2 | 97 | 53.30% | 85 | 46.70% | 492 |  |
|  | T3+T4 | 149 | 48.06% | 161 | 51.94% |  |  |
| Clinical N Status | N0 | 113 | 46.89% | 128 | 53.11% | 504 |  |
|  | N1-3 | 140 | 53.23% | 123 | 46.77% |  |  |
| Clinical M Status | M0 | 241 | 50.10% | 240 | 49.90% | 485 |  |
|  | M1 | 1 | 25.00% | 3 | 75.00% |  |  |
| Clinical Stage | Stage I+II | 60 | 50.85% | 58 | 49.15% | 494 |  |
|  | Stage III+IV | 187 | 49.73% | 189 | 50.27% |  |  |
| Surgical Margin | Negative | 175 | 51.17% | 167 | 48.83% | 447 |  |
|  | Close/Positive | 48 | 45.71% | 57 | 54.29% |  |  |
| HPV by ISH | Negative | 29 | 46.03% | 34 | 53.97% | 83 |  |
|  | Positive | 15 | 75.00% | 5 | 25.00% |  |  |
| HPV by p16 | Negative | 35 | 48.61% | 37 | 51.39% | 109 | 0.037 |
|  | Positive | 26 | 70.27% | 11 | 29.73% |  |  |
| SCC Histologic Grade | G1+G2 | 166 | 46.24% | 193 | 53.76% | 487 | 0.043 |
|  | G3+G4 | 74 | 57.81% | 54 | 42.19% |  |  |
| Lymphovascular Invasion | Negative | 113 | 51.60% | 106 | 48.40% | 339 |  |
|  | Positive | 54 | 45.00% | 66 | 55.00% |  |  |
| Perineural Invasion | Negative | 92 | 49.46% | 94 | 50.54% | 352 |  |
|  | Positive | 82 | 49.40% | 84 | 50.60% |  |  |
| Extranodal spreading of neck LN | Negative | 123 | 51.25% | 117 | 48.75% | 345 |  |
|  | Positive | 48 | 45.71% | 57 | 54.29% |  |  |
| Lympho Nodes Metastasis (H&E) | Negative | 70 | 42.42% | 95 | 57.58% | 395 | 0.047 |
|  | Positive | 119 | 51.74% | 111 | 48.26% |  |  |
| Lympho Nodes Metastasis (IHC) | Negative | 93 | 46.97% | 105 | 53.03% | 207 |  |
|  | Positive | 2 | 22.22% | 7 | 77.78% |  |  |
| Lymphnode.neck.dissection | No | 54 | 56.25% | 42 | 43.75% | 505 |  |
|  | Yes | 199 | 48.66% | 210 | 51.34% |  |  |
| Pathologic T status | T1+T2 | 96 | 53.33% | 84 | 46.67% | 446 |  |
|  | T3+T4 | 123 | 46.24% | 143 | 53.76% |  |  |
| Pathologic N status | N0 | 82 | 47.13% | 92 | 52.87% | 483 |  |
|  | N1-3 | 158 | 51.13% | 151 | 48.87% |  |  |
| Pathologic M status | M0 | 92 | 52.27% | 84 | 47.73% | 177 |  |
|  | M1 | 0 | 0.00% | 1 | 100.00% |  |  |
| Pathologic Stage | Stage I+II | 53 | 51.96% | 49 | 48.04% | 437 |  |
|  | Stage III+IV | 161 | 48.06% | 174 | 51.94% |  |  |
| Primary Therapy Outcome | Response | 60 | 49.59% | 61 | 50.41% | 137 |  |
|  | No Response | 9 | 56.25% | 7 | 43.75% |  |  |
| Followup Treatment Success | Complete Remission | 98 | 53.85% | 84 | 46.15% | 255 |  |
|  | Other | 38 | 52.05% | 35 | 47.95% |  |  |
| Adjuvant Chemotherapy | No | 53 | 47.32% | 59 | 52.68% | 172 |  |
|  | Yes | 26 | 43.33% | 34 | 56.67% |  |  |
| Smoking History | Never-Smoker | 73 | 64.04% | 41 | 35.96% | 496 | 0.003 |
|  | Smoker | 175 | 45.81% | 207 | 54.19% |  |  |
| Alcohol Drinker | less than 2U/day | 61 | 52.59% | 55 | 47.41% | 214 |  |
|  | Drinker > 2U/day | 44 | 44.90% | 54 | 55.10% |  |  |
