# Supplementary material for: AKR1C1 controls cisplatin-resistance in head and neck squamous cell carcinoma through cross-talk with the STAT1/3 signaling pathway
Source: J Exp Clin Cancer Res. 2019 Jun 10;38:245. doi: 10.1186/s13046-019-1256-2 (PMC6558898; doi:10.1186/s13046-019-1256-2)
Supplement: Supplementary file 3 — Figure S1. In vivo tumor growth abilities in HNSCC cells. Figure S2. Full length Western blot images. (DOCX 2354 kb) [file 13046_2019_1256_MOESM3_ESM.docx]

**Title: AKR1C1 controls cisplatin resistance in head and neck squamous cell carcinoma through cross-talk with the STAT1/3 signaling pathway**

Wei-Ming Chang, Yu-Chan Chang, Yi-Chieh Yang, Sze-Kwan Lin, Peter Mu-Hsin Chang, Michael Hsiao

**Supplementary Figure Legends**

**Additional file 3: Figure S1.** *In vivo* tumor growth abilities in HNSCC cells

**Additional file 3: Figure S2**. Full length Western blot images.

**Figure S1**


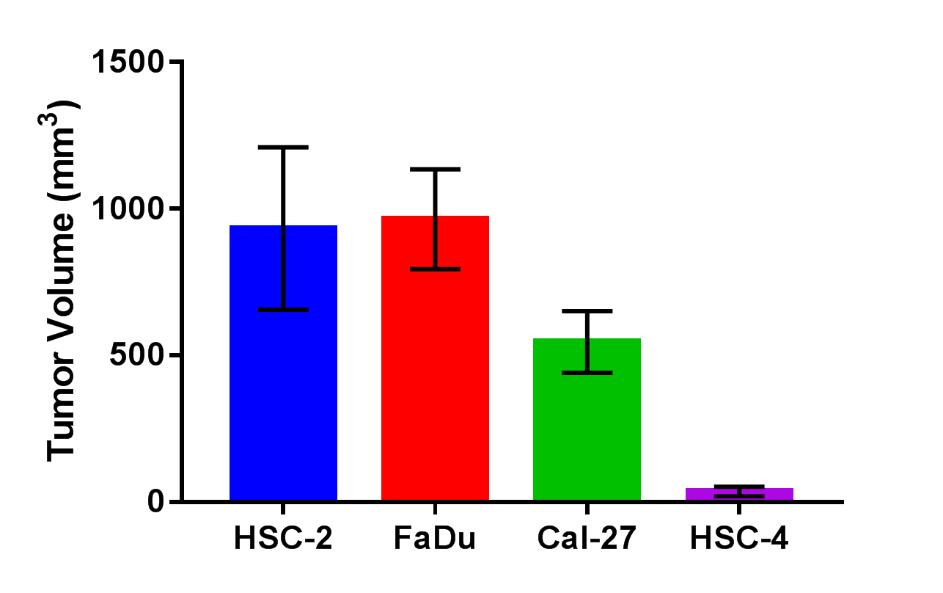


**Figure S2**


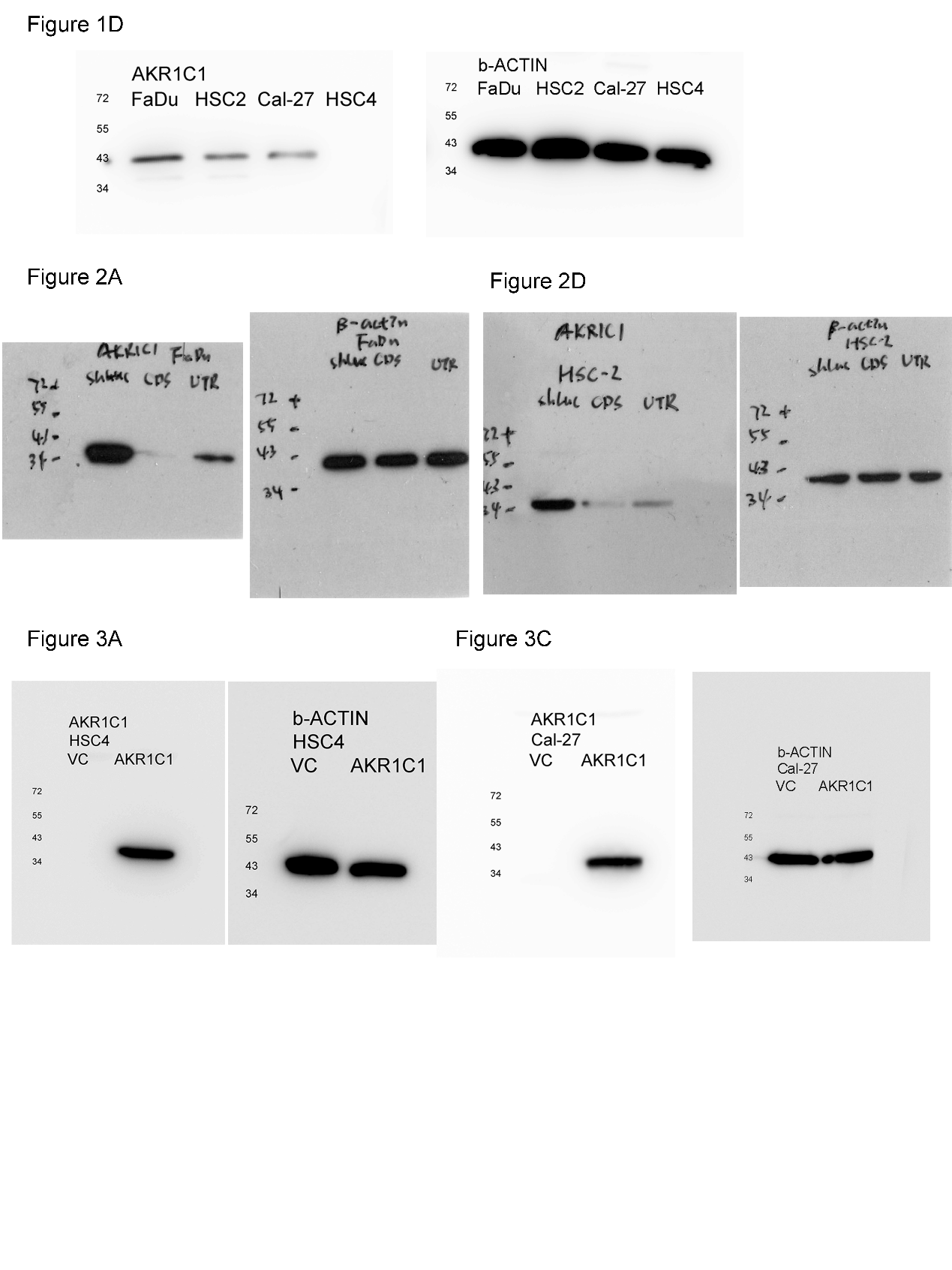


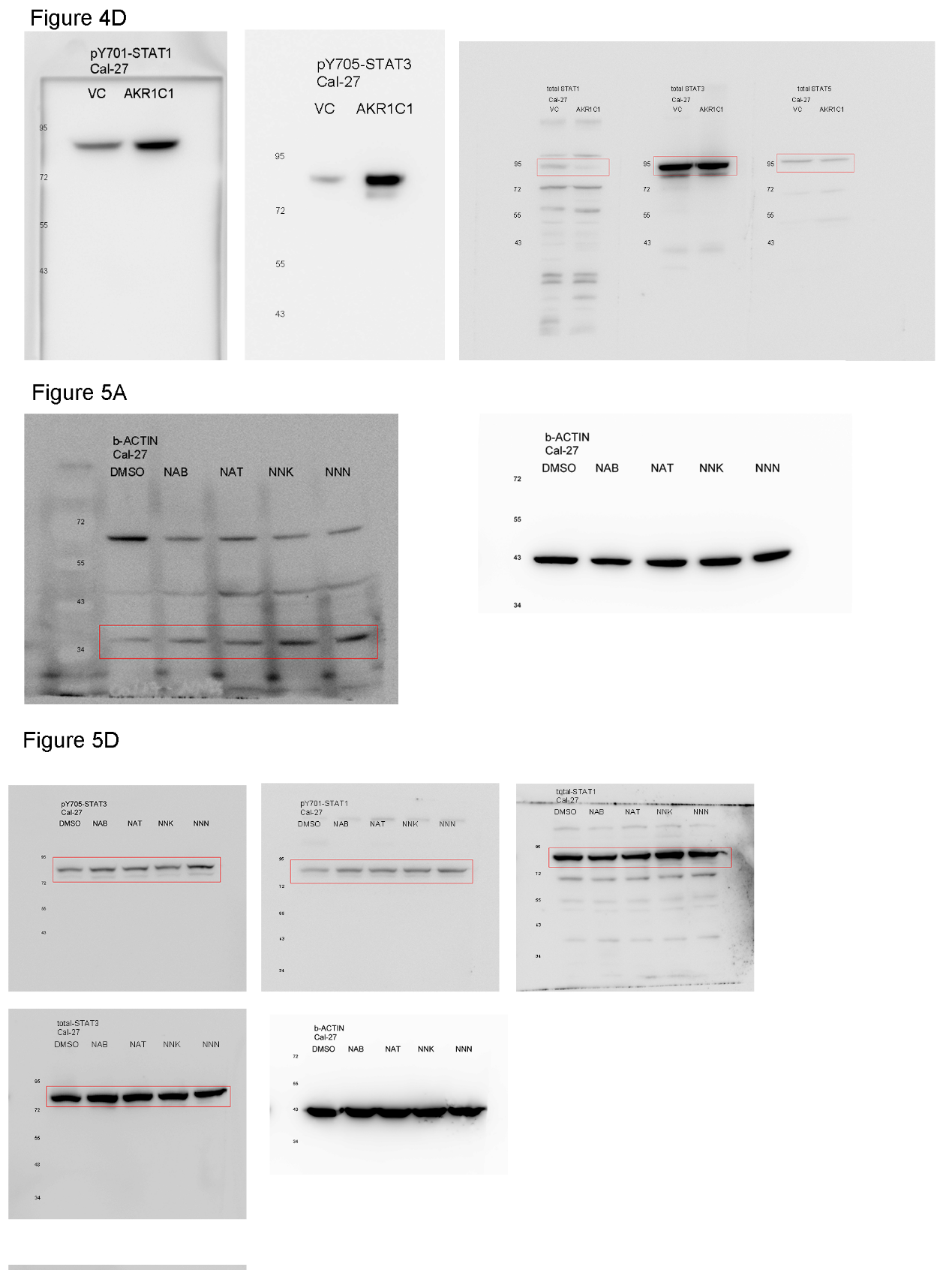

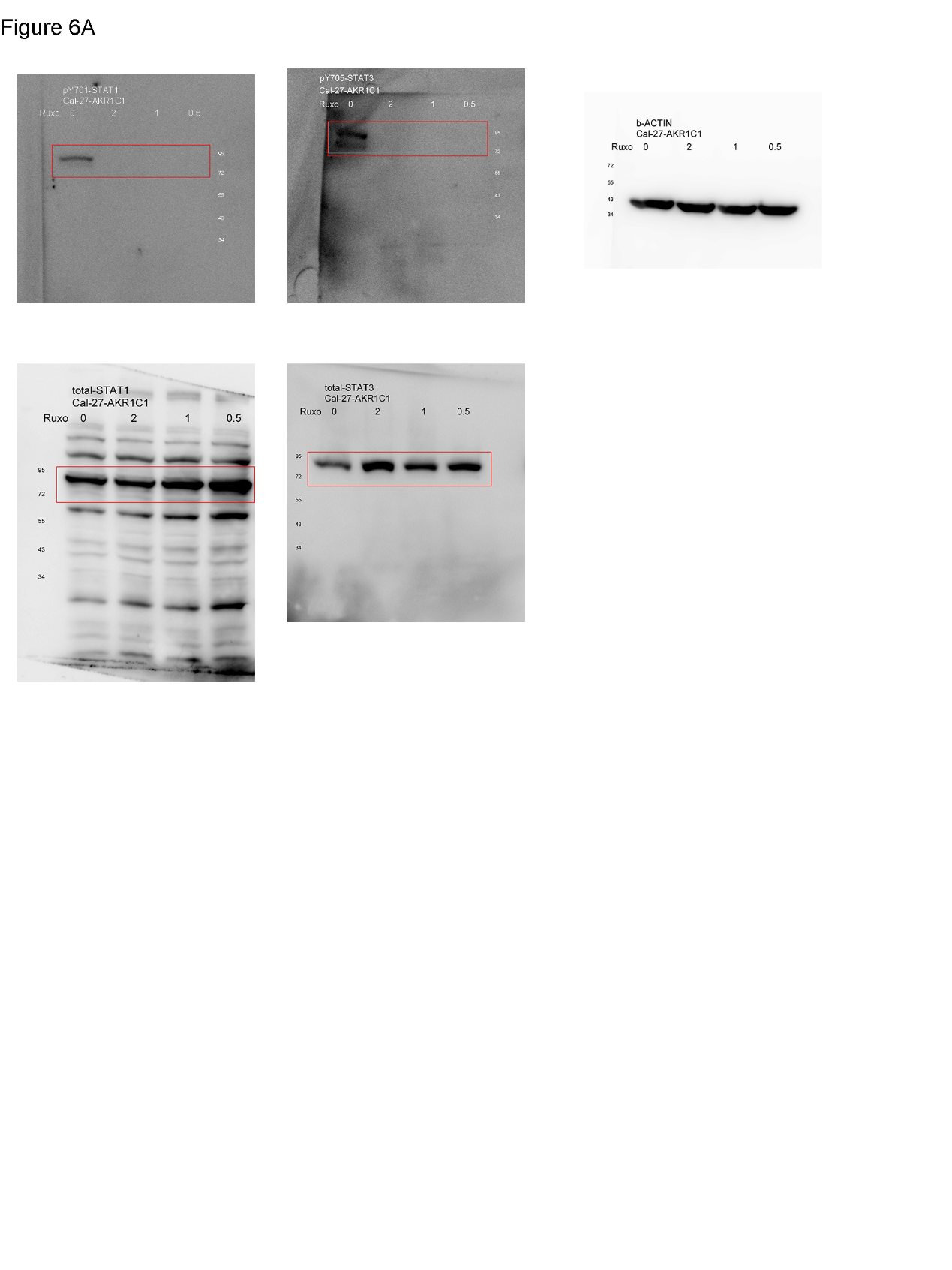


**Supplementary Table Legends**

**Supplementary Table S1.** Reagents and primer information in this manuscript

**Supplementary Table S2.** Clinical Characteristics of AKR1C1 in TCGA HNSCC cohorts

**Supplementary Table S3.** AKR1C1 controls cellular functions from Ingenuity Pathway Analysis

**Supplementary Table S4.** AKR1C1 regulates Up-stream Regulators from Ingenuity Pathway Analysis

**Supplementary Table S5.** The Up-stream Regulators of TSNAs treatment in Cal-27 cells from Ingenuity Pathway Analysis
