# Supplementary material for: AKR1C1 controls cisplatin-resistance in head and neck squamous cell carcinoma through cross-talk with the STAT1/3 signaling pathway
Source: J Exp Clin Cancer Res. 2019 Jun 10;38:245. doi: 10.1186/s13046-019-1256-2 (PMC6558898; doi:10.1186/s13046-019-1256-2)
Supplement: Supplementary file 4 — Table S3. AKR1C1 controls cellular functions from Ingenuity Pathway Analysis (DOCX 17 kb) [file 13046_2019_1256_MOESM4_ESM.docx]

| **Table S3: AKR1C1 controls cellular functions from Ingenuity Pathway Analysis** | | | | | |
| --- | --- | --- | --- | --- | --- |
| Categories | Diseases or Functions Annotation | p-Value | Activation z-score | Molecules |  |
| Cell Death and Survival | Apoptosis | 0.0279 | -1.09 | APOBEC3B,BEX2,CDK6,ITGA4,L1CAM,MIR17HG,ROR1,SPOCK1,SRPX |  |
| Cell Death and Survival | Apoptosis of tumor cell lines | 0.00379 | -0.612 | BEX2,CDK6,ITGA4,MIR17HG,ROR1,SPOCK1,SRPX |  |
| Cell Death and Survival | Necrosis | 0.0106 | -0.552 | APOBEC3B,BEX2,CDK6,ITGA4,L1CAM,MIR17HG,ROR1,RPL37A,SPOCK1,SRPX |  |
| Cell Morphology, Cellular Assembly and Organization, Cellular Function and Maintenance | Extension of cellular protrusions | 0.000212 | 0.57 | ITGA4,L1CAM,MYO5B,ROR1 |  |
| Cell-To-Cell Signaling and Interaction | Binding of tumor cell lines | 0.00297 | 0.991 | IL1R2,ITGA4,L1CAM,OXTR |  |
| Cancer, Organismal Injury and Abnormalities, Respiratory Disease | Respiratory system tumor | 0.00536 | 1.131 | APOBEC3B,CDK6,ITGA4,L1CAM,MIR17HG,MYO5B,RGS7,SRPX,TMEM27 |  |
| Cellular Development, Cellular Growth and Proliferation, Nervous System Development and Function, Tissue Development | Development of neurons | 0.01 | 1.54 | CDK6,L1CAM,MYO5B,OXTR,ROR1 |  |
| Cardiovascular System Development and Function, Organismal Development | Angiogenesis | 0.0191 | 1.698 | BEX2,ITGA4,L1CAM,MIR17HG,ROR1 |  |
| Cancer, Organismal Injury and Abnormalities | Metastasis | 0.00045 | 1.886 | AKR1C1/AKR1C2,CDK6,ITGA4,L1CAM,MIR17HG,ROR1,SRPX |  |
| Cell Death and Survival | Cell viability of tumor cell lines | 0.0497 | 1.969 | BEX2,CDK6,MIR17HG,ROR1 |  |
| Organismal Development | Size of body | 0.00237 | 2.378 | CDK6,CLMP,L1CAM,RAB3B,RGS7,TMEM27 |  |
