# Supplementary material for: AKR1C1 controls cisplatin-resistance in head and neck squamous cell carcinoma through cross-talk with the STAT1/3 signaling pathway
Source: J Exp Clin Cancer Res. 2019 Jun 10;38:245. doi: 10.1186/s13046-019-1256-2 (PMC6558898; doi:10.1186/s13046-019-1256-2)
Supplement: Supplementary file 5 — Table S4. AKR1C1 regulates Up-stream Regulators from Ingenuity Pathway Analysis (DOCX 16 kb) [file 13046_2019_1256_MOESM5_ESM.docx]

| **Table S4: AKR1C1 regulates Up-stream Regulators from Ingenuity Pathway Analysis** | | | |
| --- | --- | --- | --- |
| Upstream Regulator | Activation z-score | p-value of overlap | Target molecules in dataset |
| IL2 | 1.953 | 0.00369 | CDK6,IL1R2,ITGA4,L1CAM |
| TNF | 1.929 | 0.0483 | APOBEC3B,IL1R2,ITGA4,RGS7,SPOCK1 |
| beta-estradiol | 1.477 | 0.00353 | BEX2,COA1,IL1R2,L1CAM,MYO5B,OXTR,SPOCK1 |
| lipopolysaccharide | 1.298 | 0.0451 | APOBEC3B,IL1R2,ITGA4,RAB3B,RGS7 |
| TGFB1 | 1.109 | 0.00314 | AKR1C1/AKR1C2,ITGA4,L1CAM,PLXNC1,ROR1,SAMHD1,SPOCK1 |
| dexamethasone | 0.813 | 0.0121 | AKR1C1/AKR1C2,IL1R2,ITGA4,OXTR,ROR1,SPOCK1 |
| tretinoin | -0.175 | 0.0308 | CDK6,ITGA4,MIR17HG,ROR1,SAMHD1 |
