# Supplementary material for: AKR1C1 controls cisplatin-resistance in head and neck squamous cell carcinoma through cross-talk with the STAT1/3 signaling pathway
Source: J Exp Clin Cancer Res. 2019 Jun 10;38:245. doi: 10.1186/s13046-019-1256-2 (PMC6558898; doi:10.1186/s13046-019-1256-2)
Supplement: Supplementary file 6 — Table S5. The Up-stream Regulators of TSNAs treatment in Cal-27 cells from Ingenuity Pathway Analysis (DOCX 20 kb) [file 13046_2019_1256_MOESM6_ESM.docx]

| **Table S5: The Up-stream Regulators of TSNAs treatment in Cal-27 cells from Ingenuity Pathway Analysis** | | | |
| --- | --- | --- | --- |
| Upstream Regulator | Activation z-score | p-value of overlap | Target molecules in dataset |
| IFNG | 3.552 | 1.51E-13 | BLNK,C1R,CALB1,CTGF,CTSB,CYP24A1,DEPP1,EGLN3,EGR1,GBP2,HCAR3,HLA-DQA1,IFI27,IFI44L,IFI6,IFITM1,ITGA4,KRT14,KRT15,KRT17,MUC1,MUC4,NOTCH3,NUPR1,S100A7,SLPI,TIMP3,TP73,TREM1,TXNIP |
| TNF | 2.035 | 1.92E-10 | AQP3,ATP2B4,CTGF,CTSB,CYP1A1,DEPP1,DSC3,EGLN3,EGR1,GBP2,HCAR3,HOXA9,IFI27,IFI6,IFITM1,ITGA4,KRT15,MUC1,MUC20,MUC4,NGFR,PCDH7,RNASE4,S100A7,SAT1,SLPI,TIMP3,TREM1,TXNIP,ZNF750 |
| STAT3 | 2.621 | 9.73E-10 | CA9,CTGF,CTSB,EGR1,GBP2,HIST2H2AA3/HIST2H2AA4,HLA-DQA1,IFI27,IFI6,IFITM1,KRT17,MUC1,NDRG1,NOTCH3,S100A7,SERPINB3,SERPINB4 |
| decitabine | 3.476 | 1.62E-09 | CA9,CTGF,CYP1A1,CYP24A1,GSN,HOXA9,IFI27,IFITM1,ITGA4,KRT15,LUM,NDRG1,RNASE4,SAT1,TIMP3,TP73 |
| STAT1 | 2.216 | 8.83E-09 | C1R,EGLN3,EGR1,GBP2,HLA-DQA1,IFI27,IFI44L,IFI6,IFITM1,MUC1,MUC4,SERPINB3,SERPINB4 |
| EHF | 2.828 | 1.03E-08 | BLNK,CYP1A1,MUC1,NOTCH3,RHCG,S100A12,S100A7,SPRR1B |
| lipopolysaccharide | 4.259 | 2.44E-08 | AQP3,CEMIP,CTGF,CTSB,CYP1A1,EGR1,FYB1,GBP2,GPM6A,GSN,HCAR3,HLA-DQA1,IFI27,IFI44L,IFI6,IFITM1,ITGA4,MUC20,NGFR,NUPR1,PCDH7,S100A12,SAT1,SLPI,TIMP3,TREM1,TXNIP |
| dexamethasone | 2.484 | 0.000000248 | ATP2B4,CALB1,CTGF,CTSB,CYP1A1,CYP24A1,DEPP1,EGLN3,EGR1,GBP2,IFI6,IFITM1,ITGA4,KLHL24,KRT14,KRT17,MUC1,NGFR,OLFM4,PCSK5,RNF128,SLPI,TIMP3,TMPRSS4,TNNT1,TXNIP,ZNF750 |
| PRL | 2.571 | 0.000000607 | CTSB,EGR1,GPNMB,IFI44L,IFI6,IFITM1,KRT14,KRT15,NUPR1,RNASE4 |
| TP53 | 2.53 | 0.000000644 | AQP3,CA9,CEL,CLCA2,CTGF,CTSB,CYP1A1,CYP24A1,DSC3,EGR1,GSN,HLA-DQA1,ING3,KRT14,KRT15,NDRG1,NUPR1,PCDH7,RNASE4,SAT1,SLPI,TCEA3,TIMP3,TP73,YPEL3 |
| SMARCA4 | 2.596 | 0.000000848 | ARRDC4,ATP2B4,CTGF,CTSB,CYP1A1,EGR1,FABP6,GPM6A,IFI27,IFITM1,KRT15,LUM,MUC1,TREM1 |
| SB203580 | -3.226 | 0.00000104 | CTGF,CYP1A1,EGR1,FYB1,GBP2,HCAR3,IFI27,ITGA4,MUC4,SLPI,TIMP3,TREM1 |
| IL5 | 3 | 0.00000621 | ALDOC,CLEC2B,EGLN3,EGR1,GBP2,HCAR3,LUM,NDRG1,RASGRP2 |
| 3-deazaneplanocin | 2.2 | 0.00000885 | GPNMB,KRT17,NUPR1,SLPI,TIMP3 |
| cisplatin | 2.234 | 0.00000979 | AHNAK2,AQP3,CTGF,CTSB,EGR1,GPNMB,IFI6,IFITM1,MUC20,NOTCH3,SAT1,TIMP3,TP73,TXNIP |
| SP110 | -2.449 | 0.0000239 | AQP3,CTSB,IFI27,IFI6,IFITM1,TXNIP |
| ribavirin | 2 | 0.000038 | IFI27,IFI44L,IFI6,IFITM1 |
| PD98059 | -3.22 | 0.0000472 | CTGF,CTSB,CYP1A1,CYP24A1,EGR1,ITGA4,MUC1,RNASE4,SPRR1B,TP73,TXNIP |
| EPAS1 | 2.177 | 0.0000541 | ALDOC,CA9,CEMIP,CTGF,EGLN3,NDRG1,PTPRZ1 |
| IL13 | 2.247 | 0.0000773 | CFI,CTGF,CTSB,EGR1,GPNMB,GSN,SERPINB3,SERPINB4,SLPI |
| FGF2 | 2.369 | 0.0000951 | ANG,AQP3,CALB1,EGLN3,EGR1,GPNMB,ITGA4,NGFR,TIMP3 |
| IFNA2 | 2.598 | 0.000105 | C1R,EGLN3,GBP2,IFI27,IFI44L,IFI6,IFITM1 |
| ERK | 2.159 | 0.000113 | CTGF,CYP1A1,EGR1,MUC1,NDRG1,NOTCH3,S100A12 |
| deferoxamine | 2.373 | 0.000285 | CA9,CYP24A1,EGLN3,EGR1,IFI6,NDRG1 |
| hydrogen peroxide | 2.246 | 0.000417 | CTGF,CTSB,CYP1A1,EGR1,IFI6,MUC1,NOTCH3,TP73,TXNIP |
| STAT4 | 2.438 | 0.000547 | ALDOC,FYB1,HIST2H2AA3/HIST2H2AA4,NDRG1,RNF128,SAT1 |
| HIF1A | 2.442 | 0.000636 | ALDOC,CA9,CEMIP,CTGF,EGLN3,KRT14,MUC1,NDRG1 |
| CD38 | 2.207 | 0.000815 | ALDOC,EGLN3,GBP2,NDRG1,RASGRP2 |
| EGLN | -2.105 | 0.000838 | ALDOC,CA9,EGLN3,FABP6,RNASE4 |
| PDLIM2 | -2 | 0.000867 | CEMIP,RNF128,TXNIP,YPEL3 |
| SREBF1 | 2.433 | 0.000986 | ALDOC,CFI,DEPP1,FABP6,GPNMB,NUPR1 |
| SP1 | 2.401 | 0.00106 | CTGF,EGR1,FABP6,MUC4,NDRG1,NGFR,RASGRP2,TIMP3,TP73 |
| TRIM24 | -2 | 0.00109 | BLNK,CALB1,CYP24A1,GBP2 |
| HOXA10 | -2.236 | 0.00137 | HLA-DQA1,KRT15,RNASE4,S100A12,SAT1 |
| mifepristone | -2.4 | 0.00205 | CTGF,EGR1,ITGA4,MUC1,NDRG1,TIMP3 |
| phorbol myristate acetate | 2.505 | 0.00238 | ATP2B4,CEMIP,CTGF,CTSB,CYP1A1,CYP24A1,EGR1,HOXA9,LBH,MUC20,MUC4,SLPI,SPRR1B |
| IL6 | 2.155 | 0.00261 | ANG,CTGF,CYP1A1,EGLN3,EGR1,GBP2,HLA-DQA1,KRT14,MUC1,S100A7 |
| doxorubicin | 2.56 | 0.00277 | CTGF,CTSB,EGR1,GPNMB,NDRG1,NOTCH3,TP73 |
| NFKBIA | 2.433 | 0.00391 | CTSB,GBP2,IFI6,PCDH7,PTPRZ1,SAT1,TIMP3 |
| prednisolone | -2 | 0.00395 | CTSB,CYP1A1,ITGA4,TP73,TXNIP |
| PML | 2 | 0.00584 | IFI27,IFI44L,IFITM1,NDRG1 |
| E2F1 | 2.152 | 0.00716 | CTSB,EGR1,HIST2H2AA3/HIST2H2AA4,HOXA9,KRT14,MUC4,TP73 |
| NFkB (complex) | 2.414 | 0.00804 | C1R,CALB1,CTGF,EGR1,FABP6,GBP2,KRT17,SLPI |
| D-glucose | 2.726 | 0.0091 | ALDOC,ATP2B4,CALB1,CTGF,CTSB,EGR1,GSN,TXNIP |
| CEBPB | 2.2 | 0.0117 | BLNK,CYP1A1,CYP24A1,KRT15,NUPR1,SAT1 |
| SNCA | 2 | 0.02 | CTSB,GBP2,GSN,HLA-DQA1 |
| valproic acid | 2.236 | 0.0213 | ATP2B4,CTSB,CYP1A1,CYP24A1,FABP6,TP73 |
| methotrexate | 2 | 0.0246 | C1R,CFI,GSN,TIMP3 |
| cycloheximide | 2.089 | 0.0279 | ATP2B4,CTGF,CYP1A1,CYP24A1,EGR1 |
| LY294002 | -2.236 | 0.031 | AQP3,CA9,CTGF,EGR1,NDRG1,TREM1 |
| IL4 | 2.213 | 0.0317 | ALDOC,CTGF,GBP2,ITGA4,MUC1,NDRG1,SERPINB3,SERPINB4,TIMP3 |
